# Identification of multigene predictors of prognosis in patients with ovarian cancer

**DOI:** 10.1016/j.isci.2026.114932

**Published:** 2026-02-06

**Authors:** Xia Liu, Xiaoying Chen, Lihe Jiang, Yuting Su, Cong Wang, Zhe Du, Sanqi An, Daizheng Huang, Fuqiang Yin

**Affiliations:** 1 Key Laboratory of Longevity and Aging-related Diseases of Chinese Ministry of Education, Institute of Neuroscience and Guangxi Key Laboratory of Brain Science, School of Basic Medical Sciences, Guangxi Medical University, Nanning, Guangxi 530021, P.R. China; 2 Life Sciences Institute, Guangxi Medical University, Nanning, Guangxi 530021, P.R. China; 3 Medical Science Laboratory, Children’s Hospital, Maternal and Child Health Hospital of Guangxi Zhuang Autonomous Region, Nanning, Guangxi 530003, P.R. China; 4 School of Basic Medical Sciences, Youjiang Medical University for Nationalities, Baise, Guangxi 533000, P.R. China; 5 Key Laboratory of High-Incidence-Tumor Prevention and Treatment (Guangxi Medical University), Ministry of Education, Nanning, Guangxi 530021, P.R. China; 6 Guangxi Health Commission Key Laboratory of Basic Research on Brain Function and Disease, Guangxi Medical University, Nanning, Guangxi 530021, P.R. China; 7 Key Laboratory of Human Development and Disease Research (Guangxi Medical University), Education Department of Guangxi Zhuang Autonomous Region, Guangxi Medical University, Nanning, Guangxi 530021, P.R. China

**Keywords:** health sciences, medicine, oncology, health technology

## Abstract

The high recurrence and mortality of ovarian cancer (OC) necessitate reliable prognostic tools. As multigene models outperform single-gene markers, they are crucial for advancing clinical decision-making. From 4 microarray datasets (N = 859) and a large OC cohort (N = 1793), we identified 87 overall survival (OS)- and/or progression-free survival (PFS)-associated genes. Multigene models revealed that 25 combinations and 5 combinations predicted at least 5-year OS and 5-year PFS, respectively. Notably, compared with all combinations of 4 randomly selected genes, combinations involving *ALDH1A2*, *DCN*, *GATA6*, and *PDGFRA* were frequent among the top 20 OS models; 6 combinations predicted at least 50-month OS, and tissue microarray (155 OC samples) analyses confirmed this. Particularly, *DCN + GATA6* predicted the longest OS (90 months), with a survival difference of 3 years. Most genes correlated with immune cell abundance, especially macrophage abundance. Overall, these genes/combinations serve as valuable biomarkers to optimize OC clinical management.

## Highlights

• Multigene models outperform single genes in prognostic prediction for OC
• 25 and 5 multigene models predict at least 5-year OS and 5-year PFS, respectively
• *ALDH1A2*, *DCN*, *GATA6*, *PDGFRA*, and their combinations are novel prognostic markers for OC
• OC prognostic genes correlate with the immune microenvironment, especially macrophages

## Graphical abstract

**Figure:**
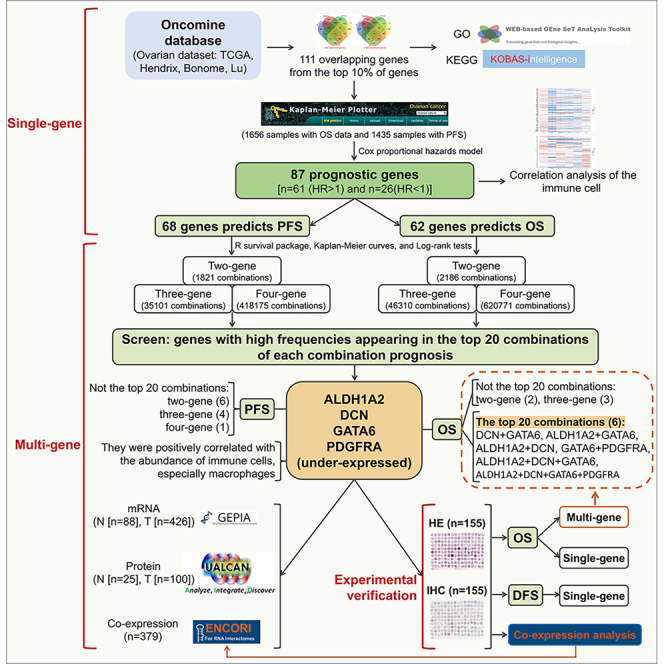

## Introduction

Ovarian cancer (OC) is a malignant tumor of the female reproductive system and had the second highest incidence and mortality in 2025.^1^ According to the latest global cancer statistics, there were more than 324,398 new cases and 206,839 deaths from OC worldwide in 2022.^2^ In China, there were 61,100 new cases and 32,600 deaths from OC in 2022.^3^ The primary treatment for OC is cytoreductive surgery combined with chemotherapy. However, OC is insidious, with high recurrence rates and 5-year survival rates of approximately 48%.^4^ Therefore, the identification of new prognostic biomarkers for adjuvant surgery combined with chemotherapy is urgently needed to prolong survival of OC patients.

Gene microarrays, high-throughput molecular techniques, and diverse bioinformatics approaches have been widely used to screen and develop effective prognostic biomarkers.^5^^,^^6^ For instance, genes associated with drug resistance and prognosis in OC have been identified through the integration of microarray data and clinical samples.^7^^,^^8^^,^^9^ Similarly, potential prognostic biomarkers and immunotherapy targets have been identified in other cancers, using big data and comprehensive bioinformatics analyses.^10^^,^^11^^,^^12^ For example, in patients with high-grade serous OC, the expression of B7-H3 and CCL2 is positively correlated with the abundance of M2 macrophages, and high B7-H3 expression is associated with a poor prognosis.^13^

Several studies have shown that the use of large sample sizes on public data platforms, the integration of new and effective mining and screening methods, and reliable experimental validation are very promising directions for the discovery of multiple effective single-gene and multigene combined tumor prognostic markers. For example, three-gene combination (*UPB1* + *SOCS2* + *RTN3*,^14^ *F2* + *GOT2* + *TRPV1*,^15^ and *GLMP* + *SLC38A6* + *WDR76*^16^) and four-gene combination (*CENPA* + *SPP1* + *MAGEB6* + *HOXD9*^17^) models can predict overall survival (OS) and disease-free survival (DFS) of patients with liver cancer. In addition, a prognostic signature-weighted combination of 21 immune-related genes achieved moderate performance in predicting 1-, 3-, and 5-year OS of OC patients, with AUCs of 0.746, 0.735, and 0.749, respectively.^18^ Understanding the molecular alterations of multiple genes other than *BRCA1*/*2* can enable more personalized diagnosis, prediction, prognosis, and treatment strategies for OC patients.^19^ A prognostic model of OC based on the characteristics of seven tumor stem cell-related genes (*GALP*, *CACNA1C*, *COL16A1*, *PENK*, *C4BPA*, *PSMA2*, and *CXCL9*) displays a significant predictive effect.^20^

On this basis, using data from four independent microarrays, namely TCGA, Hendrix, Bonome, and Lu, along with computational models and public databases, this study performed single-gene and multigene combined prognostic analyses as well as immune correlation analysis to screen for biomarkers closely related to the prognosis of OC. Importantly, multiple gene combinations and 55 newly discovered prognostic genes identified in the above large-sample data screen may be potential prognostic biomarkers for OC. In particular, the combinations related to *ALDH1A2*, *DCN*, *GATA6*, and *PDGFRA* were further validated in 155 clinical OC tissues. This study is expected to identify novel prognostic biomarkers for OC.

## Results

### Identification of 111 genes significantly dysregulated in OC

**Figure 1:**
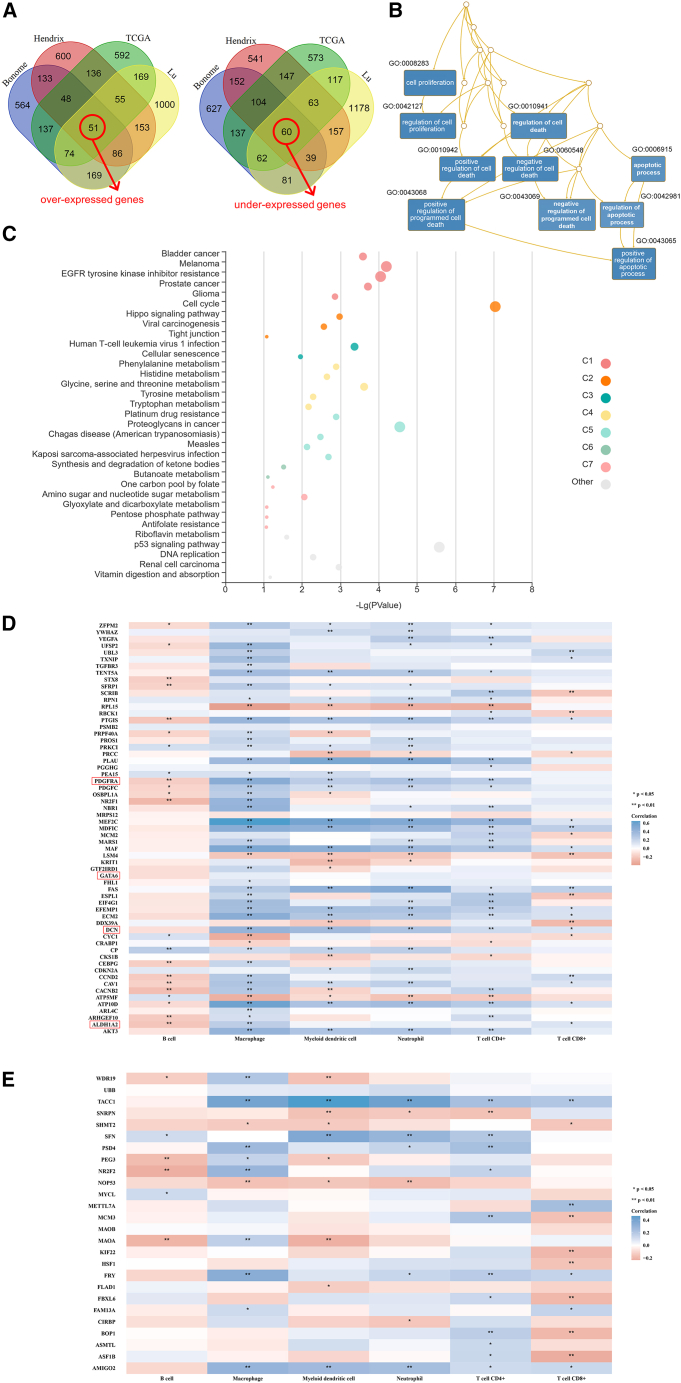
Identification of an 87-gene prognostic signature from integrated transcriptomic data and its association with the tumor immune microenvironment in ovarian cancer (A) Venn diagrams show the overlap of the top 10% dysregulated genes (over-expressed, left; under-expressed, right) across four independent microarray datasets from the Oncomine platform. TCGA, TCGA ovarian dataset; Hendrix, Hendrix ovarian dataset; Bonome, Bonome ovarian dataset; Lu, Lu ovarian dataset. (B) Gene ontology analysis of the 111 overlapping genes reveals the top 10 significantly enriched biological processes, using the WEB-based GEne SeT AnaLysis Toolkit. (C) KEGG pathway analysis of 111 overlapping genes using KOBAS-i displayed the top 5 significantly pathways with the highest enrichment ratios in each cluster. (D and E) Heatmaps depicting the correlations between the final 87 prognostic genes and the abundance of six types of tumor-infiltrating immune cells, as estimated by the TIMER algorithm and Spearman’s correlation analysis. These 87 genes are stratified into (D) 61 poor-prognosis genes (HR > 1.00) and (E) 26 favorable-prognosis genes (HR < 1.00). ∗*p* < 0.05; ∗∗*p* < 0.01. HR, hazard ratio.

Four independent microarray datasets (TCGA, Hendrix, Bonome, and Lu) from Oncomine were integrated to identify the genes dysregulated in OC (Table 1). In the TCGA, Hendrix, and Bonome ovarian datasets, 1,262 over-expressed genes and 1,262 under-expressed genes were identified as the top 10% of differentially expressed genes (DEGs). In the Lu dataset, 1,757 over-expressed and 1,757 under-expressed genes were identified as the top 10% of DEGs. Among all the top 10% of genes from these microarrays, 111 overlapping genes were identified, with 51 over-expressed genes and 60 under-expressed genes (Figure 1A). Gene ontology analysis indicated that these 111 genes might be closely related to cell death and proliferation in OC, since among the top 10 annotated biological processes (*p* < 6.37E-07, FDR < 0.00058), 8 were related to cell death or apoptosis, and 2 were related to cell proliferation (Figure 1B). KEGG pathway analysis also showed significant enrichment in pathways involved in cell death and proliferation, including cell cycle, DNA replication, cellular senescence, and so on (Figure 1C).

**Table 1:** Identification of dysregulated genes in ovarian cancer using four independent microarrays deposited in Oncomine

Microarray datasets	Type of tissues (n)	Top 10% over-expressed genes (n)	Top 10% under-expressed genes (n)
TCGA ovarian dataset	ovarian serous cystadenocarcinoma (586) vs. ovary (8)	1,262	1,262
Hendrix ovarian dataset	ovarian serous adenocarcinoma (41) vs. ovary (4)	1,262	1,262
Bonome ovarian dataset	ovarian serous carcinoma (185) vs. ovarian surface epithelium (10)	1,262	1,262
Lu ovarian dataset	ovarian serous adenocarcinoma (20) vs. ovarian surface epithelium (5)	1,757	1,757

### 87 of the 111 genes were significantly associated with the prognosis of OC patients

The relationships of the 111 genes with prognosis were analyzed in a total of 1,793 OC patients, with the subgroups of 1,656 and 1,435 samples for OS and progression-free survival (PFS) analyses, respectively. According to the log-rank tests and Cox proportional hazards regression model (Table 2), 87 of the 111 genes (78.38%) were significantly associated with prognosis (*p* < 0.05). Among them, *NR2F1* had a maximum median difference of 16.23 months in OS between the groups, and *MARS* had a maximum median difference of 10.44 months in PFS. High *DCN* expression predicted the shortest OS and PFS, with median survival times of 37.47 and 15.01 months, respectively. Low expression of *NR2F1* predicted the longest OS, with a median survival of 58.00 months. Low expression of *CEBP1* predicted the longest PFS, with a median survival of 29.03 months. Among the 51 over-expressed genes, 40 genes were associated with prognosis, with 29 genes predicting poor OS and/or PFS, and 11 genes predicting good OS and/or PFS. Among the 60 under-expressed genes, 47 were associated with prognosis, with 32 genes predicting good OS and/or PFS, and 15 genes predicting poor OS and/or PFS. In addition, a comprehensive literature search revealed that 16 of the 19 genes associated only with OS, 23 of the 25 genes associated only with PFS, and 16 of the 43 genes associated with both OS and PFS have not been reported.

**Table 2:** 87 of 111 dysregulated genes were associated with prognosis in 1,793 ovarian cancer patients in accordance with Kaplan‒Meier plot analyses

Dysregulated genes		OS		HR	PFS
		HR	*p* value		*p* value
Over-expressed genes (*n* = 40)	*LSM4*	1.30 (1.13–1.50)	2.00E-04	–	–
	*DDX39*	1.21 (1.05–1.39)	1.00E-02	–	–
	*PLAU*	1.34 (1.17–1.55)	4.10E-05	1.22 (1.08–1.39)	1.60E-03
	*MARS*	1.28 (1.13–1.46)	2.00E-04	1.44 (1.24–1.66)	1.10E-06
	*CKS1B*	1.28 (1.12–1.45)	2.00E-04	1.31 (1.15–1.49)	4.90E-05
	*PEA15*	1.27 (1.11–1.45)	4.00E-04	1.47 (1.26–1.71)	7.00E-07
	*ARL4C*	1.24 (1.09–1.41)	1.30E-03	1.44 (1.27–1.65)	3.80E-08
	*CEBPG*	1.24 (1.09–1.41)	9.00E-04	1.47 (1.27–1.71)	3.60E-07
	*MRPS12*	1.24 (1.08–1.43)	2.70E-03	1.15 (1.02–1.31)	2.77E-02
	*PRPF40A*	1.22 (1.07–1.39)	2.70E-03	1.22 (1.07–1.39)	3.70E-03
	*ESPL1*	1.22 (1.04–1.42)	1.21E-02	1.31 (1.13–1.52)	3.00E-04
	*CDKN2A*	1.20 (1.05–1.38)	8.20E-03	1.27 (1.11–1.45)	4.00E-04
	*YWHAZ*	1.18 (1.04–1.35)	1.11E-02	1.20 (1.06–1.36)	4.30E-03
	*CRABP1*	1.18 (1.02–1.36)	2.34E-02	1.16 (1.00–1.34)	4.27E-02
	*VEGFA*	1.18 (1.02–1.36)	2.71E-02	1.38 (1.19–1.59)	1.80E-05
	*PRCC*	1.17 (1.02–1.35)	2.82E-02	1.41 (1.23–1.61)	6.10E-07
	*KRIT1*	1.16 (1.02–1.32)	2.33E-02	1.38 (1.22–1.57)	6.30E-07
	*MCM2*	1.16 (1.00–1.35)	4.89E-02	1.17 (1.02–1.36)	3.02E-02
	*PRKCI*	–	–	1.38 (1.21–1.57)	1.00E-06
	*SCRIB*	–	–	1.34 (1.15–1.55)	1.30E-04
	*RBCK1*	–	–	1.29(1.11–1.51)	1.20E-03
	*ATP5J2*	–	–	1.29(1.11–1.49)	7.00E-04
	*EIF4G1*	–	–	1.23 (1.09–1.40)	1.10E-03
	*CYC1*	–	–	1.21 (1.07–1.38)	3.30E-03
	*PSMB2*	–	–	1.21 (1.06–1.38)	5.70E-03
	*GTF2IRD1*	–	–	1.19 (1.02–1.38)	2.51E-02
	*CP*	–	–	1.18 (1.04–1.34)	1.05E-02
	*RPN1*	–	–	1.18 (1.03–1.35)	1.49E-02
	*ATHL1*	–	–	1.17 (1.03–1.34)	1.68E-02
	*PSD4*	0.76 (0.66–0.88)	3.00E-04	–	–
	*MCM3*	0.82 (0.71–0.93)	3.00E-03	–	–
	*SHMT2*	0.82 (0.71–0.94)	6.20E-03	–	–
	*KIF22*	0.82 (0.72–0.93)	2.70E-03	–	–
	*BOP1*	0.82 (0.72–0.94)	3.30E-03	–	–
	*FBXL6*	0.83 (0.72–0.96)	1.05E-02	–	–
	*FLAD1*	0.86 (0.75–0.97)	1.82E-02	–	–
	*HSF1*	0.79 (0.69–0.91)	1.00E-03	0.78 (0.68–0.89)	3.00E-04
	*MYCL1*	0.82 (0.72–0.94)	3.30E-03	0.84 (0.74–0.96)	7.70E-03
	*SFN*	–	–	0.78 (0.68–0.90)	4.00E-04
	*ASF1B*	–	–	0.85 (0.74–0.98)	2.20E-02
Under-expressed genes (*n* = 47)	*ECM2*	1.30 (1.13–1.49)	2.00E-04	–	–
	*OSBPL1A*	1.24 (1.07–1.43)	3.30E-03	–	–
	*RPL15*	1.20 (1.04–1.39)	1.34E-02	–	–
	*STX8*	1.16 (1.00–1.35)	4.68E-02	–	–
	*SFRP1*	1.16 (1.01–1.34)	4.20E-02	–	–
	*DCN*	1.42 (1.23–1.64)	2.10E-06	1.44 (1.27–1.64)	1.60E-08
	*NR2F1*	1.42 (1.22–1.66)	8.50E-06	1.40 (1.22–1.61)	9.50E-07
	*GATA6*	1.41 (1.23–1.63)	1.50E-06	1.34 (1.17–1.53)	1.60E-05
	*ALDH1A2*	1.38 (1.20–1.58)	3.30E-06	1.41 (1.22–1.63)	2.70E-06
	*PDGFRA*	1.32 (1.14–1.53)	2.00E-04	1.34 (1.16–1.56)	9.50E-05
	*EFEMP1*	1.31 (1.15–1.50)	4.40E-05	1.42 (1.24–1.63)	2.40E-07
	*TGFBR3*	1.27 (1.11–1.46)	6.00E-04	1.19 (1.03–1.37)	1.66E-02
	*MAF*	1.27 (1.10–1.48)	1.10E-03	1.37 (1.20–1.56)	2.10E-06
	*MEF2C*	1.27 (1.10–1.46)	9.00E-04	1.41 (1.23–1.61)	3.70E-07
	*UBL3*	1.26 (1.09–1.46)	1.70E-03	1.28 (1.12–1.46)	2.00E-04
	*FHL1*	1.26 (1.09–1.44)	1.30E-03	1.48 (1.29–1.69)	8.80E-09
	*CAV1*	1.24 (1.09–1.41)	1.00E-03	1.18 (1.04–1.35)	9.50E-03
	*PTGIS*	1.23 (1.08–1.40)	2.00E-03	1.26 (1.11–1.43)	3.00E-04
	*MDFIC*	1.22 (1.06–1.40)	6.20E-03	1.14 (1.00–1.29)	5.00E-02
	*AKT3*	1.21 (1.06–1.37)	4.20E-03	1.35 (1.19–1.54)	3.50E-06
	*FAS*	1.17 (1.01–1.35)	3.56E-02	1.27 (1.10–1.47)	1.10E-03
	*ARHGEF10*	1.15 (1.00–1.31)	4.34E-02	1.32 (1.14–1.53)	2.00E-04
	*ZFPM2*	1.14 (1.00–1.30)	4.25E-02	1.34 (1.19–1.52)	3.60E-06
	*NBR1*	–	–	1.37 (1.19–1.58)	1.90E-05
	*PROS1*	–	–	1.37 (1.19–1.57)	6.40E-06
	*UFSP2*	–	–	1.30 (1.14–1.48)	8.10E-05
	*FAM46A*	–	–	1.28 (1.12–1.46)	3.00E-04
	*TXNIP*	–	–	1.23 (1.07–1.42)	3.90E-03
	*PDGFC*	–	–	1.20 (1.06–1.37)	5.00E-03
	*ATP10D*	–	–	1.20 (1.05–1.37)	6.40E-03
	*CACNB2*	–	–	1.20 (1.05–1.35)	5.30E-03
	*CCND2*	–	–	1.19 (1.05–1.35)	6.50E-03
	*SNRPN*	0.80 (0.70–0.92)	1.40E-03	–	–
	*CIRBP*	0.82 (0.72–0.94)	5.00E-03	–	–
	*WDR19*	0.82 (0.72–0.94)	3.70E-03	–	–
	*FRY*	0.83 (0.73–0.94)	4.10E-03	–	–
	*TACC1*	0.87 (0.75–1.00)	4.67E-02	–	–
	*FAM13A*	0.79 (0.69–0.91)	9.50E-04	0.86 (0.74–0.99)	4.25E-02
	*UBB*	0.81 (0.71–0.92)	1.60E-03	0.77 (0.68–0.87)	3.60E-05
	*METTL7A*	0.82 (0.72–0.94)	4.30E-03	0.87 (0.77–0.99)	3.08E-02
	*ASMTL*	0.83 (0.72–0.94)	4.60E-03	0.85 (0.74–0.99)	3.26E-02
	*AMIGO2*	0.85 (0.74–0.98)	2.24E-02	0.78 (0.67–0.91)	1.30E-03
	*MAOB*	0.85 (0.75–0.97)	1.33E-02	0.83 (0.73–0.94)	4.50E-03
	*NR2F2*	0.86 (0.76–0.98)	2.48E-02	0.86 (0.74–0.99)	3.71E-02
	*MAOA*	–	–	0.78 (0.67–0.90)	7.50E-04
	*GLTSCR2*	–	–	0.85 (0.74–0.97)	1.55E-02
	*PEG3*	–	–	0.87 (0.75–0.99)	4.02E-02

OS, overall survival; PFS, progression-free survival; HR, hazard ratio. The expression of all genes was dichotomized into high and low values, using the autoselected best cut-off by the Kaplan‒Meier plotter. Statistical tests: Kaplan-Meier survival analysis with log-rank test and Cox proportional hazards model.

### Two-gene combination prognostic model

**Figure 2:**
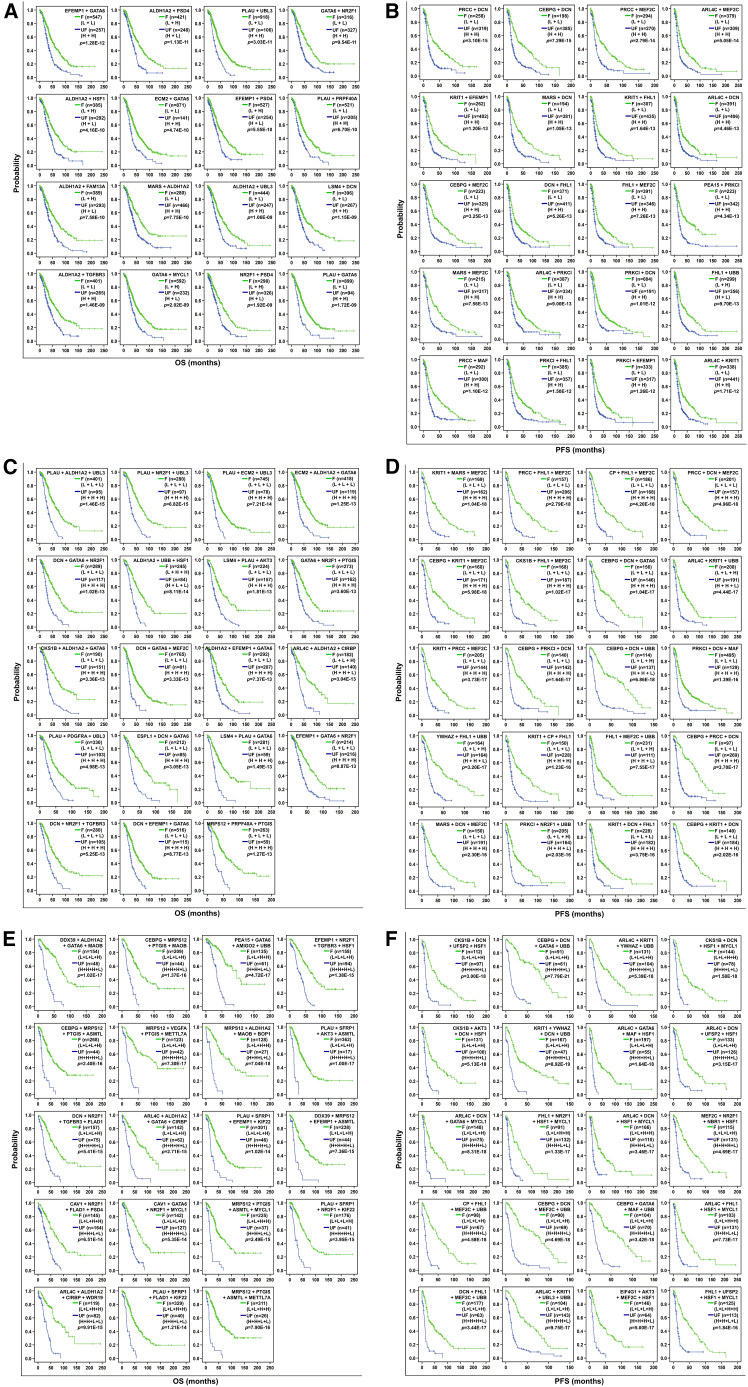
Kaplan-Meier analysis of the associations of two-gene, three-gene, and four-gene combination expression patterns with OS and PFS in ovarian cancer patients (A) Survival curves for 16 of the top 20 two-gene combinations with the smallest *p* values for OS. Patients were stratified based on two-gene expression patterns: the favorable expression group (F, green line) with longer survival, and the unfavorable expression group (UF, blue line) with shorter survival. The remaining 4 combinations are shown in Figure 3A. H, high expression; L, low expression. Statistical test: Kaplan-Meier survival analysis with log-rank test, all combinations with *p* < 0.001. (B) Survival curves for the top 20 two-gene combinations with the smallest *p* values for PFS. Grouping and curve definitions are the same as in (A). (C) Survival curves for 19 of the top 20 three-gene combinations with the smallest *p* values for OS. Grouping and curve definitions are the same as in (A). The remaining 1 combination is shown in Figure 3A. (D) Survival curves for the top 20 three-gene combinations with the smallest *p* values for PFS. Grouping and curve definitions are the same as in (A). (E) Survival curves for 19 of the top 20 four-gene combinations with the smallest *p* values for OS. Grouping and curve definitions are the same as in (A). The remaining 1 combination is shown in Figure 3A. (F) Survival curves for the top 20 four-gene combinations with the smallest *p* values for PFS. Grouping and curve definitions are the same as in (A). OS, overall survival; PFS, progression-free survival.

**Figure 3:**
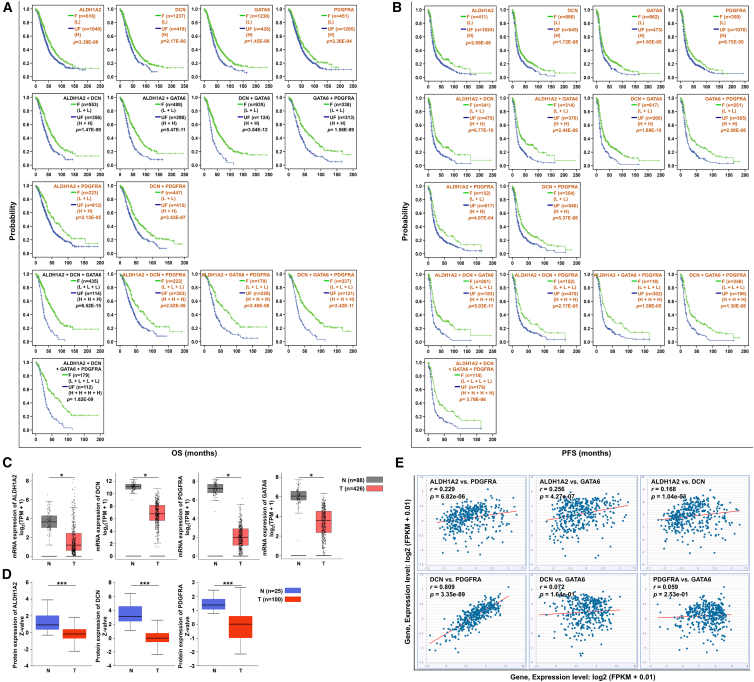
Analysis of the association of *ALDH1A2*, *DCN*, *GATA6*, *PDGFRA,* and their combinations with prognosis and expression in ovarian cancer (A) Kaplan-Meier analysis of individual genes and their combinations with OS. Patients were stratified based on gene expression patterns: the favorable expression group (F, green line) with longer survival, and the unfavorable expression group (UF, blue line) with shorter survival. Genes highlighted in black are those present in the top 20 OS-related combinations. H, high expression; L, low expression. Statistical test: Kaplan-Meier survival analysis with log-rank test. (B) Kaplan-Meier analysis of individual genes and their combinations with PFS. Grouping and curve definitions are the same as in (A). (C) Differential mRNA expression of *ALDH1A2*, *DCN*, *GATA6*, and *PDGFRA* between 88 normal ovarian tissues (N) and 426 ovarian cancer tissues (T) from the GEPIA database, displayed on a log2(TPM+1) scale. Statistical test: Mann-Whitney U test. ∗*p* < 0.05. (D) Differential protein expression of *ALDH1A2*, *DCN*, *GATA6*, and *PDGFRA* between 25 normal ovarian tissues (N) and 100 ovarian cancer tissues (T) from UALCAN, with Z values representing standard deviations from the median. Statistical test: Student’s *t* test. ∗∗∗*p* < 0.001. (E) Pairwise co-expression analysis of *ALDH1A2*, *DCN*, *GATA6*, and *PDGFRA* in 379 ovarian cancer cases from StarBase. Gene expression values are scaled as log_2_(FPKM+0.01); r, correlation coefficient. OS, overall survival; PFS, progression-free survival; GEPIA, gene expression profiling interactive analysis; UALCAN, University of Alabama at Birmingham Cancer Data Analysis Portal. Box plots show the median (line), interquartile range (box), and Tukey's whiskers (extending to 1.5×interquartile range).

Given the strong associations between these 87 genes and clinical outcomes in OC, the effect of the multigene combination on prognosis was further evaluated using a similar approach according to previous studies.^21^^,^^22^ First, by using a combination of 2 genes from the 62 genes related to OS, we found that a total of 1,821 combinations were related to OS (*p* < 0.01), and the top 20 combinations with the smallest *p* values contained 17 genes, as shown in Figures 2A and 3A and Table S3. *GATA6* + *NR2F1* showed the maximum median difference of 26.87 months in OS between patients with “favorable” and “unfavorable” expressions. The median survival time of the combination *GATA6* + *NR2F1* (L + L) with “favorable” expression was 61.00 months. In contrast, the median survival time of the combination *GATA6* + *NR2F1* (H + H) with “unfavorable” expression was 34.13 months. The “favorable” expression of the combination *MARS* + *ALDH1A2* (L + L) predicted the longest OS, with a median survival of 62.50 months. The “unfavorable” expression of the combination *PLAU* + *UBL3* (H + H) predicted the shortest OS, with a median survival of 26.23 months.

A total of 2,186 two-gene combinations from the 68 genes related to PFS were identified (*p* < 0.01), and the top 20 combinations with the smallest *p* values contained 13 genes, as shown in Figure 2B and Table S4. *PEA15* + *PRKCI* resulted in the maximum median difference of 25.08 months in PFS between the “favorable” and “unfavorable” expression groups. The “favorable” expression of the combination *PEA15* + *PRKCI* (L + L) predicted the longest PFS, with a median survival of 42.58 months. In contrast, the median survival time of the combination *PEA15* + *PRKCI* (H + H) with “unfavorable” expression was 17.50 months. The “unfavorable” expression of the combination *PRKCI* + *DCN* (H + H) predicted the shortest PFS, with a median survival of 13.20 months.

### Three-gene combination prognostic model

A total of 35,101 three-gene combinations from the 62 genes related to OS were identified (*p* < 0.01), and the top 20 combinations with the smallest *p* values contained 22 genes, as shown in Figures 2C and 3A and Table S5. The median difference in OS between the “favorable” and “unfavorable” expression groups was 41.67 months for *CKS1B* + *ALDH1A2* + *GATA6*. The “favorable” expression of the combination *CKS1B* + *ALDH1A2* + *GATA6* (L + L + L) predicted the longest OS, with a median survival of 73.17 months. In contrast, the median survival time of the combination *CKS1B* + *ALDH1A2* + *GATA6* (H + H + H) with “unfavorable” expression was 31.50 months. The “unfavorable” expression of the combination *DCN* + *GATA6* + *MEF2C* (H + H + H) predicted the shortest OS, with a median survival time of 22.97 months.

A total of 46,310 three-gene combinations from the 68 genes related to PFS were identified (*p* < 0.01), and the top 20 combinations with the smallest *p* values contained 16 genes, as shown in Figure 2D and Table S6. *CEBPG* + *DCN* + *UBB* resulted in a maximum median difference of 56.88 months in PFS between the “favorable” and “unfavorable” expression groups. The “favorable” expression of the combination *CEBPG* + *DCN* + *UBB* (L + L + H) predicted the longest PFS, with a median survival of 69.11 months. In contrast, the median survival time of the combination *CEBPG* + *DCN* + *UBB* (H + H + L) with “unfavorable” expression was 12.23 months. The “unfavorable” expression of the combination *FHL1* + *MEF2C* + *UBB* (H + H + L) predicted the shortest PFS, with a median survival time of 11.92 months.

### Four-gene combination prognostic model

A total of 418,175 four-gene combinations from the 62 genes related to OS were identified (*p* < 0.01), and the top 20 combinations with the smallest *p* values contained 31 genes, as shown in Figures 2E and 3A and Table S7. The maximum difference in OS between the “favorable” and “unfavorable” expression groups was 78.00 months for *MRPS12* + *VEGFA* + *PTGIS* + *METTL7A*. The “favorable” expression of the two combinations *MRPS12* + *VEGFA* + *PTGIS* + *METTL7A* (L + L + L + H) and *PEA15* + *GATA6* + *AMIGO2* + *UBB* (L + L + H + H) predicted the longest OS, with a median survival time of 95.00 months. In contrast, the median survival time for patients with respect to the combination *MRPS12* + *VEGFA* + *PTGIS* + *METTL7A* (H + H + H + L) with “unfavorable” expression was 17.00 months. The “unfavorable” expression of the combination *MRPS12* + *PTGIS* + *ASMTL* + *METTL7A* (H + H + L + L) predicted the shortest OS, with a median survival time of 14.00 months.

A total of 620,771 four-gene combinations from the 68 genes related to PFS were identified (*p* < 0.01), and the top 20 combinations with the smallest *p* values contained 20 genes, as shown in Figure 2F and Table S8. *CEBPG* + *DCN* + *GATA6* + *UBB* resulted in a maximum median difference of 68.64 months in PFS between the “favorable” and “unfavorable” expressions. The “favorable” expression of the combination *CEBPG* + *DCN* + *GATA6* + *UBB* (L + L + L + H) predicted the longest PFS, with a median survival time of 79.57 months. In contrast, the median survival time of the combination *CEBPG* + *DCN* + *GATA6* + *UBB* (H + H + H + L) with “unfavorable” expression was 10.93 months. The “unfavorable” expression of the combination *DCN* + *FHL1* + *MEF2C* + *UBB* (H + H + H + L) predicted the shortest PFS, with a median survival time of 10.00 months.

### The multigene prognostic model resulted in a more significant difference in median survival compared with the single-gene prognostic model

Overall, we noticed that the months of the difference in median survival between the groups increased when the genes were combined, and the greater the number of genes combined, the higher the difference in median survival. Among the top 20 genes related to OS of OC patients, 17 had median survival difference of 6–12 months, and the median survival difference for 3 genes, *NR2F1* (16.23 months), *ALDH1A2* (15.20 months), and *PLAU* (13.06 months), surpassed 12 months (Tables 2 and 3). When the genes were combined, for the top 20 two-gene, three-gene, and four-gene combination prognostic models, the differences in median OS were mainly distributed between 1 and 24 months (75.00%, 15/20), 24 and 48 months (95.00%, 19/20), and 36 and 48 months (55.00%, 11/20), respectively. Overall, among all the combinations, the greatest difference in survival difference of ≥60 months was associated with 3 four-gene combinations, including *MRPS12* + *VEGFA* + *PTGIS* + *METTL7A*, *ARL4C* + *ALDH1A2* + *GATA6* + *CIRBP*, and *PEA15* + *GATA6* + *AMIGO2* + *UBB* (Tables 3 and S7), and the favorable expression of 5 two-gene combinations, 9 three-gene combinations, and 11 four-gene combinations predicted a survival time of more than 5 years (≥60 months) (Tables S3, S5, and S7).

**Table 3:** Summary of differences in the median survival of ovarian cancer patients from 87 single-gene and the top 20 combination prognostic models

Prognostic models (top 20)		Median survival difference (months)							
		6–12	12–18	18–24	24–30	30–36	36–48	48–60	≧60
OS	1 gene	17	3	–	–	–	–	–	–
	2-gene	–	–	15	5	–	–	–	–
	3-gene	–	–	1	6	7	6	–	–
	4-gene	–	–	–	1	2	11	3	3
PFS	1 gene	20	–	–	–	–	–	–	–
	2-gene	–	12	6	2	–	–	–	–
	3-gene	–	1	3	5	8	1	2	–
	4-gene	–	–	3	3	8	3	1	2

OS, overall survival; PFS, progression-free survival; n, number of prognostic models. 2-gene, 3-gene, and 4-gene indicate two-gene combinations, three-gene combinations, and four-gene combinations, respectively.

Among the top 20 genes related to PFS, all had a median survival difference of 6–12 months (Tables 2 and 3). When the genes were combined, for the top 20 two-gene, three-gene, and four-gene combination prognostic models, the PFS times of the median survival differences were distributed mainly in the ranges of 12–18 months (60.00%, 12/20), 30–36 months (40.00%, 8/20), and 30–36 months (40.00%, 8/20), respectively. Collectively, among all the combinations, the greatest difference in median survival (≥60 months) was observed for 2 four-gene combinations, including *CEBPG* + *DCN* + *GATA6* + *UBB* and *CEBPG* + *DCN* + *MEF2C* + *UBB* (Tables 3 and S8), and the favorable expression of 2 three-gene combinations and 3 four-gene combinations predicted a survival time of more than 5 years (≥60 months) (Tables S4, S6, and S8).

### 87 prognostic genes were associated with immune cells in OC

The 87-gene prognostic signature was further investigated for its relationship with the tumor immune microenvironment. The 61 poor prognosis genes (hazard ratio [HR] > 1.00) were significantly associated with a myeloid-enriched, immunosuppressive landscape, showing strong positive correlations with macrophages and neutrophils, alongside negative correlations with B cells (Figure 1D). Notably, despite concurrent positive correlations with CD8^+^ T cells and dendritic cells for a gene subset (Figure 1D), the dominant pattern suggests a milieu where myeloid-driven suppression may override cytotoxic function. Conversely, the 26 favorable prognosis genes (HR < 1.00) defined a divergent context, showing a significant positive correlation with CD4^+^ T cells but a negative correlation with CD8^+^ T cells and no association with macrophages (Figure 1E). This pattern implies a protective mechanism potentially mediated by a CD4^+^ T cell-driven response, independent of conventional CD8^+^ T cell cytotoxicity.

### *ALDH1A2*, *DCN*, *GATA6*, *PDGFRA*, and their combinations had specific prognostic value in OC

Among the top 20 four-gene combinations significantly associated with OS (*p* < 0.001) (Figures 2E and 3A; Table S7), the member genes of the combination *ALDH1A2* + *DCN* + *GATA6* + *PDGFRA*, the lower expression of which significantly predicted longer OS and PFS (*p* < 0.001) (Figure 3B), also frequently appeared in the top 20 two-gene and three-gene combinations related to OS (*p* < 0.001), including 4 two-gene combinations (*DCN* + *GATA6*, *ALDH1A2* + *GATA6*, *ALDH1A2* + *DCN*, and *GATA6* + *PDGFRA*) and 1 three-gene combination (*ALDH1A2* + *DCN* + *GATA6*). In addition, the combinations of the 4 genes excluded from the top 20 genes were also significantly associated with OS (*p* < 0.001), which included 2 two-gene combinations (*ALDH1A2* + *PDGFRA* and *DCN* + *PDGFRA*) and 3 three-gene combinations (*ALDH1A2* + *DCN* + *PDGFRA*, *ALDH1A2* + *GATA6* + *PDGFRA*, and *DCN* + *GATA6* + *PDGFRA*) (Figure 3A). All these results indicated that the 4 genes *ALDH1A2*, *DCN*, *GATA6*, and *PDGFRA*, as well as their two-, three-, and four-gene combinations, were significantly related to OS. Favorable expression of all their combinations among the top 20 predicted OS of at least 50 months. Additionally, the PFS models of the two-, three-, and four-gene combinations of these 4 genes did not appear in the top 20 combinations, but they were also significantly associated with PFS (*p* < 0.001) (Figure 3B). Furthermore, the expression of the four genes *ALDH1A2*, *DCN*, *GATA6*, and *PDGFRA* was positively correlated with the abundance of most tumor immune cells. Notably, the expression of all four genes was most strongly and positively correlated with the abundance of macrophages (Figure 1D). Therefore, these four genes were selected for further analysis.

### Low expression of *ALDH1A2*, *DCN*, *GATA6*, *PDGFRA*, and their combinations correlated with longer OS in OC patients

**Figure 4:**
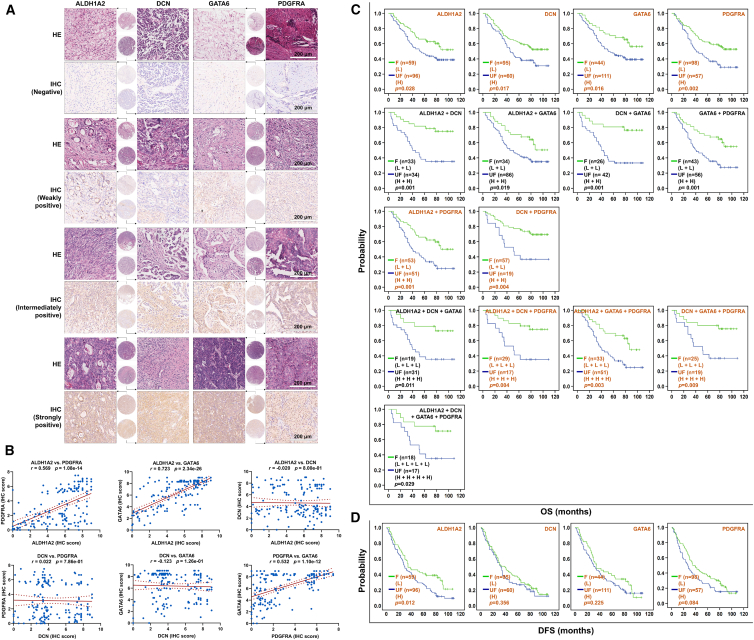
Analysis of *ALDH1A2*, *DCN*, *GATA6*, and *PDGFRA* protein expression based on IHC results and their association with ovarian cancer prognosis (A) Representative H&E and IHC staining images showing strong positive, moderately positive, weakly positive, and negative protein expression of *ALDH1A2*, *DCN*, *GATA6*, and *PDGFRA* in 155 ovarian cancer tissues. H&E, hematoxylin and eosin; IHC, immunohistochemistry. Scale bars: 200 μm. (B) Pairwise expression correlations of *ALDH1A2*, *DCN*, *GATA6*, and *PDGFRA* in 155 ovarian cancer tissues analyzed by Spearman’s correlation analysis. r, correlation coefficient. (C and D) Kaplan-Meier curves analyzing the association of individual genes and their combinations with OS and DFS based on IHC data from 155 ovarian cancer tissues. IHC scores of <5 were defined as low expression (corresponding to negative/weakly positive), while scores ≥ 5 were defined as high expression (corresponding to moderately/strongly positive). Patients were stratified by gene combination expression patterns into the favorable expression group (F, green line) with longer survival and the unfavorable expression group (UF, blue line) with shorter survival. Combinations marked in black are consistent with the top 20 OS-related predictions. H, high expression; L, low expression. Statistical test: Kaplan-Meier survival analysis with log-rank test. OS, overall survival; DFS, disease-free survival.

As mentioned above, *ALDH1A2*, *DCN*, *GATA6*, and *PDGFRA* under-expressed in OC tissues according to four independent microarrays (Table 1). Consistent with these results, we found that the mRNA expression of the 4 genes was significantly downregulated in 426 OC tissues compared with 88 normal control tissues (*p* < 0.05) (Figure 3C), and the protein expression was downregulated in 100 OC tissues compared with 25 normal control tissues (*p* < 0.001), although the protein data for *GATA6* are not available (Figure 3D). Co-expression analysis revealed that these four genes positively co-expressed in OC. Specifically, *ALDH1A2* positively co-expressed with *DCN*, *GATA6*, and *PDGFRA* in 379 OC tissues, and *DCN* co-expressed with *PDGFRA*, although the positive co-expression of *GATA6* with *DCN* and *PDGFRA* was not statistically significant (Figure 3E). These results were further confirmed on the basis of 155 OC tissue array samples. Representative immunohistochemistry (IHC) images of negative, weakly positive, intermediately positive, and strongly positive expression of the 4 proteins and corresponding hematoxylin and eosin (H&E)-stained OC samples are shown in Figure 4A. On the basis of the IHC results, we found that the combinations *ALDH1A2* and *PDGFRA*, *ALDH1A* and *GATA6*, and *PDGFRA* and *GATA6* positively co-expressed in 155 OC samples (Figure 4B), although the co-expression of the remaining combinations was not detected.

Moreover, prognostic analyses revealed that patients with low expression of *ALDH1A2*, *DCN*, *GATA6*, and *PDGFRA* had a survival probability greater than 50% and the mean survival differences of 13.88–18.27 months, indicating good OS (*p* < 0.05) (Figure 4C and Table S9). When the four genes were combined, for the 6 two-gene combinations, 4 three-gene combinations, and 1 four-gene combination prognostic models, the mean OS times were 18.76–35.06 months, 22.01–32.15 months, and 25.87 months, respectively (*p* < 0.05) (Figure 4C and Table S9). The “favorable” expression of these combination models yielded a survival probability of higher than 50%, except for the *ALDH1A2* + *GATA6* + *PDGFRA* combination (Figure 4C and Table S9).

Among the 6 two-gene combination prognostic models, the combinations *ALDH1A2* + *DCN* and *DCN* + *GATA6* yielded the greatest difference in mean survival time (32.81 and 35.05 months, respectively), and the greatest mean survival time for “favorable” expression of the combination *DCN* + *GATA6* (L + L) was 90.15 months (Figure 4C and Table S9). Among the 4 three-gene combination prognostic models, *DCN* + *GATA6* + *PDGFRA*, *ALDH1A2* + *DCN* + *GATA6*, and *ALDH1A2* + *DCN* + *PDGFRA* had the greatest mean survival time differences of 30.16, 31.61, and 32.14 months, respectively; in particularly, the greatest mean survival time for the “favorable” expression of the *ALDH1A2* + *DCN* + *PDGFRA* (L + L + L) combination was 90.38 months (Figure 4C and Table S9). With respect to the four-gene combination *ALDH1A2* + *DCN* + *GATA6* + *PDGFRA*, an average survival time of 84.10 months was predicted by “favorable” expression (Figure 4C and Table S9). The above results are similar to those predicted by the GSE26193 dataset (Table S12). Additionally, low *ALDH1A2* expression was associated with longer DFS in OC patients (*p* < 0.05) (Figure 4D and Table S10). Low expression of *DCN, GATA6*, and *PDGFRA* also increased DFS in OC patients, although the differences were not statistically significant (Figure 4D and Table S10).

## Discussion

OC arises in the deep pelvic cavity and often presents with no overt clinical manifestations in its early stages. It is characterized by high malignancy and associated with a dismally low 5-year survival rate.^1^^,^^4^ Moreover, the prognostic outcomes of OC treatment are intricately influenced by factors such as chemotherapy responsiveness and the tumor immune microenvironment.^23^^,^^24^ Therefore, the identification of robust prognostic biomarkers to guide adjuvant therapy for OC represents a critical strategy to improve the long-term survival of OC patients.^25^ In fact, a large number of gene expression signatures have been developed for survival prediction and, consequently, for improving treatment, providing guidance for personalized treatment decisions. However, multiple studies have reported that the median survival time of patients for whom a single gene is predicted is generally less than 5 years.^8^^,^^26^^,^^27^^,^^28^ In OC, the median OS and/or PFS predicted by *CKS1B*,^29^ *ALDH1A2*,^30^^,^^31^ *GATA6*,^32^ *DCN*,^33^^,^^34^^,^^35^ *UBB*,^36^ *MRPS12*,^37^ and *PEA15*^38^ are less than 5 years.

Previous studies have reported that compared with single-gene prognostic models, multigene prognostic models have greater predictive value for tumors.^14^^,^^15^^,^^16^^,^^17^ In OC, similar studies have been performed, and several multigene prognostic models have been constructed. For example, we previously identified pairs of genes that are positively co-expressed in OC tissues, such as *SLC7A11* with *STX17* or *UVRAG*; *FHL1* with *FLNC*, *CAV1*, *PPP1R12B*, or *FLNA*; and *NCALD* with *CX3CL1*.^8^^,^^9^^,^^27^ Among them, the combination of *SLC7A11* with *STX17* was reported to be a better predictor of OS and PFS than either of the two genes,^8^ and the combination of *SLC7A11* with *UVRAG* was found to be a stronger predictor of OS and post-progression survival (PPS).^8^ The combination of *FHL1* with *FLNC*, *CAV1*, *PPP1R12B*, or *FLNA* provided better OS and PPS prediction than any gene alone,^27^ and the combination of *NCALD* and *CX3CL1* provided better OS, PFS, and PPS predictions than any single gene.^9^ In this study, based on our identified 87 genes associated with OS and/or PFS, two-gene, three-gene, and four-gene prognostic models were constructed, and a much better prognostic effect was observed, specifically for the top 20 combination models. For the OS prognostic models, five of the top 20 two-gene combinations had median survival time exceeding 5 years in the “favorable” expression group of each combination, with the combination *MARS* + *ALDH1A2* (L + L) predicting the longest survival time of 62.50 months. Among the top 20 three-gene combinations, 9 combinations had median survival time exceeding 5 years in the “favorable” expression group. Specifically, the combination *CKS1B* + *ALDH1A2* + *GATA6* (L + L + L) predicted the longest survival time (73.17 months). Eleven of the top 20 four-gene combinations had median survival time exceeding 5 years in the “favorable” expression group of each combination, and the *MRPS12* + *VEGFA* + *PTGIS* + *METTL7A* (L + L + L + H) and *PEA15* + *GATA6* + *AMIGO2* + *UBB* (L + L + H + H) combinations both predicted the longest OS time—up to 95.00 months. Taken together, a total of 25 multigene combinations could predict OS of more than 5 years; specifically, 2 of these combinations could predict an OS duration of up to 95 months (nearly 8 years). Similarly, for the PFS prognostic models, a total of 5 multigene combinations (2 of three-gene combinations and 3 of four-gene combinations) could predict survival times exceeding 5 years; specifically, the combination *CEBPG* + *DCN* + *GATA6* + *UBB* (L + L + L + H) predicted the longest PFS time—up to 79.57 months. Interestingly, 3 combinations (*MRPS12* + *VEGFA* + *PTGIS* + *METTL7A*, *PEA15* + *GATA6* + *AMIGO2* + *UBB*, and *ARL4C* + *ALDH1A2* + *GATA6* + *CIRBP*) predicted median OS differences of more than 5 years between “favorable” and “unfavorable” expression, whereas 2 combinations (*CEBPG* + *DCN* + *GATA6* + *UBB* and *CEBPG* + *DCN* + *MEF2C* + *UBB*) predicted median PFS differences of more than 5 years.

Notably, compared with all 11 combinations of 4 randomly selected genes, the combination of *ALDH1A2*, *DCN*, *GATA6*, and *PDGFRA* appeared most frequently among the top 20 combinations related to OS. These included 6 specific combinations: *ALDH1A2* + *DCN* + *GATA6* + *PDGFRA*, *ALDH1A2* + *DCN* + *GATA6*, *DCN* + *GATA6*, *ALDH1A2* + *GATA*, *ALDH1A2* + *DCN*, and *GATA6* + *PDGFRA*. The “favorable” expression of these 6 combinations predicted an OS time of at least 50 months. These combinations were also significantly associated with PFS in OC patients. Several previous studies have shown that low levels of ALDH1A2, DCN, GATA6, and PDGFRA are favorable prognostic factors for cancer.^39^^,^^40^^,^^41^^,^^42^^,^^43^^,^^44^^,^^45^ In OC, a few studies have indicated that *ALDH1A2*, *DCN*, *GATA6*, and *PDGFRA* are potentially associated with patients’ survival. We previously reported that a high expression of *ALDH1A2* contributes to OC invasion and metastasis and predicts poor outcomes in OC patients.^30^ High expression of *DCN* is closely related to a reduced survival rate in OC patients,^33^^,^^34^^,^^35^ and high levels of *PDGFRA* are associated with high tumor grade, a high proliferation index, and poor patient outcome.^46^ *GATA6* is an independent risk factor for the prognosis of OC patients,^47^ and miR-10a-5p can inhibit the proliferation, tumorigenicity, migration, and invasion of OC cells through the downregulation of *GATA6* expression.^32^ Consistent with the findings of previous studies, our results indicated that *ALDH1A2*, *DCN*, *GATA6*, and *PDGFRA* were downregulated in OC tissues and that low levels of the 4 genes predicted longer OS in OC patients based on an open data study and verification in 155 cases of OC tissue samples from commercial tissue microarrays. Moreover, the free combination of these four genes offered better prognostic potential than the individual combinations of these genes. All 11 combinations of these 4 genes predicted an OS time of at least 75 months, with the combination *DCN* + *GATA6* predicting the longest OS time of up to 90 months and median OS differences of almost 3 years between “favorable” and “unfavorable” expression, thus representing the best difference among the 11 genes.

In addition, this study provides a potential candidate gene pool for subsequent prognostic biomarker research. A total of 87 genes potentially related to survival of OC patients were identified, and the findings are essentially consistent with those of previous studies. For example, high expression of *NR2F1*^48^ and *CEBPG*^49^ was associated with poor OS and PFS in OC patients, respectively. Our results indicated that low expression of *NR2F1* predicted 58.00 months of OS in OC patients, and low expression of *CEBPG* predicted 29.03 months of PFS. Besides, among the 43 genes associated with both OS and PFS in OC patients, 16 (*PLAU*, *MARS*, *PRPF40A*, *PRCC*, *KRIT1*, *MYCL1*, *MAF*, *MEF2C*, *UBL3*, *MDFIC*, *ARHGEF10*, *FAM13A*, *METTL7A*, *ASMTL*, *MAOB*, and *NR2F2*) have not been reported previously. Similarly, among the 19 genes associated only with OS, 16 (*LSM4*, *DDX39*, *PSD4*, *SHMT2*, *KIF22*, *BOP1*, *FLAD1*, *ECM2*, *OSBPL1A*, *RPL15*, *STX8*, *SNRPN*, *CIRBP*, *WDR19*, *FRY*, and *TACC1*) have not been reported, and among the 25 genes associated only with PFS, 23 (*PRKCI*, *SCRIB*, *RBCK1*, *ATP5J2*, *EIF4G1*, *CYC1*, *PSMB2*, *GTF2IRD1*, *CP*, *RPN1*, *ATHL1*, *ASF1B*, *NBR1*, *PROS1*, *UFSP2*, *FAM46A*, *PDGFC*, *ATP10D*, *CACNB2*, *CCND2*, *MAOA*, *GLTSCR2*, and *PEG3*) have not been reported. Taken together, the associations of a total of 55 genes with survival of OC patients remain unreported, and these genes may be potential prognostic biomarkers.

Previous studies have suggested that prognosis-related genes are significantly involved in regulating the tumor immune microenvironment.^50^^,^^51^^,^^52^^,^^53^ For example, the co-expression of *VISTA*/*CTLA4*/*PD1* in tumor cells and the expression of 21 immune genes reflecting the immune cell status and infiltration level are associated with a favorable immune microenvironment and better prognosis for OC patients.^18^^,^^54^ Similarly, in this study, the majority of the 61 poor prognosis genes (HR > 1.00) showed strong positive correlations with myeloid cells (macrophages and neutrophils) but negative correlations with B cells. Among them, *ALDH1A2*, *DCN*, and *PDGFRA* were all significantly positively correlated with macrophages. When myeloid cells dominate, they may potentially suppress the cytotoxic function of T cells. Consistent with this, other studies have reported that the recruitment or infiltration of macrophages and neutrophils promotes tumor progression and recurrence.^55^^,^^56^ When genes related to poor prognosis are positively correlated with the infiltration levels of immune cells such as macrophages, neutrophils, and T cells, the abundance and infiltration level of M2 macrophages will significantly increase in the high-risk group.^57^^,^^58^ Moreover, an increased abundance of tumor-associated macrophages is a predictor of poor prognosis in patients with bladder cancer, breast cancer, OC, and low-grade glioma,^59^^,^^60^ and a high expression of genes positively correlated with M2-like tumor-associated macrophage infiltration contributes to poor prognosis in OC patients.^61^ In contrast, among the 26 favorable prognosis genes (HR < 1.00) in this study, most showed significant positive correlations with CD4^+^ T cells and negative correlations with CD8^+^ T cells, while exhibiting no association with macrophages. Furthermore, studies have indicated that direct tumor recognition by CD4^+^ T cells can effectively mediate tumor growth suppression and coordinate anti-tumor immune responses.^62^ Collectively, these findings suggest that this protective pattern may be primarily driven by a CD4^+^ T cell-dependent mechanism.

In summary, a total of 87 genes related to OS and PFS were identified, and the associations of 55 genes with OC prognosis have not been reported. The relationships of two-gene, three-gene, and four-gene free combinations of these 87 genes with prognosis were investigated, and 25 combinations that predicted OS of at least 5 years were determined. Notably, compared with all 11 combinations of 4 randomly selected genes, the combination of *ALDH1A2*, *DCN*, *GATA6*, and *PDGFRA* appeared most frequently among the top 20 combinations related to OS, and all 11 combinations predicted an OS time of at least 75 months. Especially the combination *DCN* + *GATA6*, with the longest predicted OS time of up to 90 months, predicted median OS differences of almost 3 years between “favorable” and “unfavorable” expression groups. All of these genes and their combinations could be potential prognostic biomarkers for the management of patients with OC.

### Limitations of the study

Our study has several limitations. First, it relies on a retrospective analysis of public datasets and commercial tissue microarrays, carrying risks of selection bias and uncontrollable confounding factors that may compromise model robustness. Additionally, heterogeneity may exist among public datasets due to differences in sample collection, detection platforms, and follow-up protocols. Furthermore, although we verified key findings in 155 tissue samples (from a single center), multicenter, prospective validation and functional experiments are required in future studies.

## Resource availability

### Lead contact

Requests for further information and resources should be directed to and will be fulfilled by the lead contact, Fuqiang Yin (yinfq@mail2.sysu.edu.cn).

### Materials availability

The commercial ovarian cancer tissue microarray (HOvaC160Su01) used in this study is available from Shanghai Outdo Biotech Co., Ltd. The H&E/IHC staining images and IHC scoring data are provided in the supplemental information.

### Data and code availability

• This paper analyzes existing, publicly available data, accessible at Oncomine database (https://www.oncomine.org/resource/login.html), Kaplan-Meier Plotter (https://kmplot.com/analysis/index.php?p=service&cancer=ovar), The Cancer Genome Atlas (https://portal.gdc.cancer.gov/), Gene Expression Profiling Interactive Analysis (http://gepia.cancer-pku.cn/), University of Alabama at Birmingham Cancer Data Analysis Portal (http://ualcan.path.uab.edu/), and StarBase v3.0 (http://starbase.sysu.edu.cn/).
• This paper does not report original code.
• The derivative data generated from the commercial tissue microarray, including de-identified clinical information, H&E/IHC staining images, IHC staining scores, and inter-observer consistency data, are fully provided in the supplemental information. Any additional information required to reanalyze the data reported in this paper is available from the lead contact upon request.

## Acknowledgments

This work was supported by Guangxi Natural Science Foundation (2024GXNSFDA010045), the 10.13039/501100001809National Natural Science Foundation of China (82260721 and 81903644), the Guangxi Science and Technology Program under grant (AD25069077), and the grant of research project on high-level talents of Youjiang Medical College for Nationalities (grant no. YY2021SK02).

## Author contributions

F.Y. and X.L. designed and reviewed the study; X.C. and F.Y. drafted the manuscript; X.C., F.Y., and D.H. revised the manuscript; F.Y., D.H., L.J., and S.A. carried out the majority of the bioinformatics analysis; X.C. carried out the majority of the experiments; F.Y., X.L., X.C., Y.S., C.W., and Z.D. participated in material preparation and data collection. All authors have read and approved the final manuscript.

## Declaration of interests

The authors declare no competing interests.

## STAR★Methods

### Key resources table

**Table:** 

REAGENT or RESOURCE	SOURCE	IDENTIFIER
**Antibodies**		

ALDH1A2 antibody	Abcam	Cat# ab96060; RRID: AB_10679336
Anti-Decorin antibody	Abcam	Cat# ab151988; RRID: AB_2915927
GATA6 Polyclonal Antibody	Thermo Fisher Scientific	Cat# PA1-104; RRID: AB_2539879
Anti-PDGFR alpha antibody [EPR5480]	Abcam	Cat# ab134123; RRID: AB_3739751

**Biological samples**		

Ovarian cancer tissue microarray	Shanghai Outdo Biotech Co., Ltd; This paper	HOvaC160Su01; Tables S1, S2, S11 and Data S1

**Chemicals, peptides, and recombinant proteins**		

Xylene	Tianjin Aopusheng Chemical Co., Ltd.	JN-EJB-500
Anhydrous ethanol	Shanghai Sangon Biotech Co., Ltd.	A500737
Neutral gum	Beijing Solarbio Science & Technology Co., Ltd.	G8590
Citrate repair solution	Beijing Zhongshan Golden Bridge Biotechnology Co., Ltd.	ZLI-9065
PBS phosphate buffer dry powder	Beijing Solarbio Science & Technology Co., Ltd.	P1010
Triton X-100	Shanghai Biyuntian Biotechnology Co., Ltd.	P096
Blocking goat serum	Beijing Solarbio Science & Technology Co., Ltd.	SL038
Hematoxylin staining solution	Beijing Zhongshan Golden Bridge Biotechnology Co., Ltd.	BSBA-4021A

**Critical commercial assays**		

Hematoxylin and eosin staining kit	Shanghai Biyuntian Biotechnology Co., Ltd.	C0105S
Hydrochloric acid ethanol rapid differentiation solution	Shanghai Biyuntian Biotechnology Co., Ltd.	C0163M
Universal two-step method kit (mouse/rabbit enhanced polymer detection system)	Beijing Zhongshan Golden Bridge Biotechnology Co., Ltd.	PV-9000
DAB chromogenic kit	Wuhan Saiweier Biological Technology Co., Ltd.	G1212

**Deposited data**		

Microarray datasets	Oncomine database	https://www.oncomine.org/resource/login.html
Ovarian cancer prognosis data	Kaplan‒Meier Plotter	https://kmplot.com/analysis/index.php?p=service&cancer=ovar
RNA-sequenced expression profiling (level 3) and corresponding clinical data for ovarian cancer	TCGA dataset	https://portal.gdc.cancer.gov/
mRNA expression of ALDH1A2, DCN, PDGFRA, and GATA6	GEPIA	http://gepia.cancer-pku.cn/
Protein expression of ALDH1A2, DCN, and PDGFRA	UALCAN	http://ualcan.path.uab.edu/
Correlation analysis of ALDH1A2, DCN, GATA6, and PDGFRA	StarBase v3.0	http://starbase.sysu.edu.cn/

**Software and algorithms**		

R (version 4.1.0)	Posit	https://www.r-project.org/
SPSS (version 26.0)	IBM	https://www.ibm.com/support/pages/downloading-ibm-spss-statistics-26-end-support-30-sep-2025

**Other**		

Gene Ontology Analysis Website	The WEB-based GEne SeT AnaLysis Toolkit	https://www.webgestalt.org/
KEGG Pathway Enrichment Analysis Website	KOBAS-i	http://kobas.cbi.pku.edu.cn
Immune-related analysis website	ASSISTANT for Clinical Bioinformatics	https://www.aclbi.com/static/index.html#/

### Experimental model and study participant details

#### Patient samples

The OC tissue microarray (HOvaC160Su01) purchased from Shanghai Outdo Biotech Co., Ltd. was approved by the Ethics Committee of Shanghai Outdo Biotech Co., Ltd. (No. YB M-05-02). Informed consent was obtained from all participants from the Shanghai Outdo Biotech Co., Ltd. (Shanghai, China). The Medical Ethics Committee of Guangxi Medical University has approved the study. Tissue microarray of OC confirmed by formalin-fixed, paraffin-embedded, Hematoxylin‒eosin staining and immunohistochemistry. This OC tissue microarray contains 155 OC tissues with clinical data for OS and DFS. The OC tissue sources included were all female patients, with an age range of 20–75 years. Ovaries belong to the reproductive organs of female animals. The effect of age on the results of the study was not analyzed in detail in this study, which warrants further investigation. The clinical characteristics and IHC scoring data of the 155 patients with OC were summarized in Tables S1 and S2.

### Method details

#### Acquisition, annotation, and pathway enrichment of dysregulated gene data

The TCGA ovarian dataset, Hendrix ovarian dataset, Bonome ovarian dataset, and Lu ovarian dataset were downloaded from the Oncomine database (https://www.oncomine.org/resource/login.html).^63^ The top 10% of genes with dysregulated expression in the 4 OC microarrays were selected for intersection analysis. The WEB-based GEne SeT AnaLysis Toolkit (https://www.webgestalt.org/)^64^ was used to annotate the biological processes of the dysregulated genes. KEGG pathway enrichment analysis of differentially expressed genes was performed using KOBAS-i (http://kobas.cbi.pku.edu.cn).^65^ Each bubble represents an enriched function, and the size of the bubble is from small to large: [0.05,1], [0.01,0.05), [0.001,0.01), [0.0001,0.001), [1e-10,0.0001), [0,1e-10). For each cluster, if there are more than 5 terms, the top 5 with the highest enrichment ratio will be displayed.

#### Computational profiling of gene signatures and systematic literature review

Data from 1,793 OC patients were downloaded from the Kaplan‒Meier Plotter (https://kmplot.com/analysis/index.php?p=service&cancer=ovar).^66^ This dataset includes 565 cases from the TCGA ovarian cohort and 14 independent microarrays from the GEO profiles. The 14 microarrays represent GSE14764 (*n* = 80), GSE15622 (*n* = 35), GSE18520 (*n* = 63), GSE19829 (*n* = 28), GSE23554 (*n* = 28), GSE26193 (*n* = 107), GSE26712 (*n* = 195), GSE27651 (*n* = 49), GSE30161 (*n* = 58), GSE3149 (*n* = 116), GSE51373 (*n* = 28), GSE63885 (*n* = 101), GSE65986 (*n* = 55), and GSE9891 (*n* = 285). The aforementioned multiple datasets have undergone normalized integration within the Kaplan-Meier Plotter database.^66^ Among these, 1,656 patients had clinical data for OS, and 1,435 had clinical data for PFS. In the prognostic analysis of single gene expression patterns, the expression levels of all genes were divided into high and low values using the autoselected best cutoff provided by the Kaplan‒Meier Plotter.^67^ Kaplan‒Meier estimation of survival functions and log-rank tests were used to evaluate the effect of genes on DFS and OS. A Cox proportional hazards model was used for multivariate analysis of OC prognosis. Survival analyses were performed using the R survival package (version 3.2–7) in R (version 4.1.0). The Kaplan‒Meier survival curves and Cox proportional hazards regression model for DFS and OS were generated by IBM SPSS (version 26.0). Furthermore, the prognostic value of the combined expression patterns of 2, 3, and 4 genes was evaluated using methods similar to those in previous studies.^21^^,^^22^

Referring to previous search strategies,^16^^,^^68^ a full-scale literature search was performed in PubMed (up to April 16, 2025) by using the following terms: (“Gene official symbol” OR “Official full name”) AND (“Ovarian cancer” OR “Ovarian neoplasms”[MeSH Terms]) AND (“Overall survival” OR “Progression-free Survival” OR “Prognosis” OR” Prognostic” OR “Survival” OR “Outcome”) AND ((((((((((((((((((((1990/01/01:2025/04/16[Date - Publication] NOT “retraction of publication”[Publication Type]) NOT “newspaper article”[Publication Type]) NOT “Editorial”[Publication Type]) NOT “Bibliography”[Publication Type]) NOT “News”[Publication Type]) NOT “Directory”[Publication Type]) NOT “Autobiography”[Publication Type]) NOT “practice guideline”[Publication Type]) NOT “published erratum”[Publication Type]) NOT “Guideline”[Publication Type]) NOT “video audio media”[Publication Type]) NOT “Interview”[Publication Type]) NOT “retracted publication”[Publication Type]) NOT “Biography”[Publication Type]) NOT “historical article”[Publication Type]) NOT “comment”[Publication Type]) NOT “commentary”[Title]) NOT “comment”[Title]) NOT “Erratum”[Title]) NOT “Retracted”[Title]). Publications were considered eligible if they met the following criteria: studies examining the association of target gene expression levels with OS and/or PFS in OC patients.

#### Correlation analysis of gene expression and immune cell

RNA-sequenced expression profiling (level 3) and corresponding clinical data for OC were downloaded from the TCGA dataset (https://portal.gdc.cancer.gov/). The pheatmap package (version 1.0.12; https://CRAN.R-project.org/package=pheatmap) in R (version 4.0.3) was used to visualize multiple genes associated with B cells, macrophages, myeloid dendritic cells, neutrophils, CD4^+^ T cells, and CD8^+^ T cells in OC.^69^ Correlations between quantitative variables without a normal distribution were analyzed using Spearman correlation analysis. *p* < 0.05 was considered to indicate statistical significance.

#### Bioinformatics analysis of gene expression levels

The mRNA expression of *ALDH1A2*, *DCN*, *PDGFRA*, and *GATA6* in 88 normal ovarian tissues and 426 OC tissues was analyzed using Gene Expression Profiling Interactive Analysis (GEPIA, http://gepia.cancer-pku.cn/).^70^ The protein expression of *ALDH1A2*, *DCN*, and *PDGFRA* in 25 normal ovarian tissues and 100 primary OC tissues was analyzed using the University of Alabama at Birmingham Cancer Data Analysis Portal (UALCAN, http://ualcan.path.uab.edu/).^71^ The correlation between the expression of *ALDH1A2*, *DCN*, *GATA6*, and *PDGFRA* was examined by analyzing 379 OC samples from the StarBase v3.0 project (http://starbase.sysu.edu.cn/).^72^

#### H&E and IHC staining

OC tissue identification was performed using H&E standard staining according to previous methods.^73^ Tissue samples were processed in the following order: dewaxing with xylene, dehydration with ethanol, staining with haematoxylin solution, dehydration with ethanol, staining with eosin, dehydration with ethanol, clearing with xylene, and sealing with neutral tree adhesive.

The protein levels of *ALDH1A2*, *DCN*, *GATA6*, and *PDGFRA* in OC tissues were measured by IHC. The standard IHC procedure followed previously reported protocols.^74^ Tissue samples were processed in the following order: xylene dewaxing, ethanol dehydration, citric acid antigen repair, hydrogen peroxide block endogenous peroxidase, drop primary antibody, 4 °C wet box incubation overnight, 37 °C box rewarming, drop secondary antibody, diaminobenzidine color development, redyeing with haematoxylin, hydrochloric acid ethanol differentiation, tap water anti-blueing, and dehydration seal. The primary antibodies and dilutions used were as follows: ALDH1A2 (ab96060; Abcam), dilution 1:300; DCN (ab151988; Abcam), dilution 1:2000; GATA6 (PA1-104; Invitrogen), dilution 1:500; and PDGFRA (ab134123; Abcam), dilution 1:50. Tissue section staining was independently assessed by three pathologists (Kappa>0.75, *p* < 0.001, Table S11) using the immunoreactive score standard.^75^^,^^76^ The percentage of tumor cell staining was scored from 3 to 0: 3 (75%–100%), 2 (50%–75%), 1 (25%–50%), and 0 (0%–25%). The intensity of staining was scored from 0 to 3: 0: negative, 1: weak, 2: moderate, and 3: strong. The final immunostaining score (range, 0 to 9) was calculated by multiplying the percentage score and the intensity score. A final immunostaining score of <5 was defined as “low expression”, and a score of ≥5 was defined as “high expression”. Prognostic analysis of single-gene and combined-gene models was performed using the same methods as those used for the aforementioned survival analysis.

### Quantification and statistical analysis

Statistical analyses and data graphing were carried out by R (version 4.1.0) and IBM SPSS (version 26.0) unless otherwise stated. Statistical tests, exact *n* values, definitions of n for each experiment, and dispersion and precision measures were provided in the legends, results, or tables. The Kaplan‒Meier survival function, log-rank test, and Cox proportional hazards model were used to evaluate the prognostic value of single gene and/or combined gene expression patterns in survival analysis. Spearman correlation analysis was used to analyze the coexpression of the two genes and the correlation between gene expression levels and immune cell abundance. Comparisons between two groups were conducted using the Mann-Whitney U test or Student’s *t* test. Fleiss' Kappa and Cohen’s Kappa were used to assess agreement among all three evaluators and between each pair of evaluators, respectively. Statistically significant differences were indicated by *p* < 0.05 (∗*p* < 0.05, ∗∗*p* < 0.01, ∗∗∗*p* < 0.001).

## Supplemental information

Document S1. Tables S2–S12

Table S1. The clinical information and IHC scoring data of ovarian tissue microarray (HOvaC160Su01)

Data S1. Information on HOvaC160Su01 Supplemental information for HOvaC160Su01 includes an array list, an H&E staining result image, and IHC staining result images for *ALDH1A2*, *DCN*, *GATA6*, and *PDGFRA*.
