# Supplementary material for: Identification of multigene predictors of prognosis in patients with ovarian cancer
Source: iScience. 2026 Feb 6;29(3):114932. doi: 10.1016/j.isci.2026.114932 (PMC12936820; doi:10.1016/j.isci.2026.114932)
Supplement: Document S1. Tables S2–S12 [file mmc1.pdf]

iScience, Volume *29*

Supplemental information

Identification of multigene predictors of prognosis in patients with ovarian cancer

Xia Liu, Xiaoying Chen, Lihe Jiang, Yuting Su, Cong Wang, Zhe Du, Sanqi An, Daizheng Huang, and Fuqiang Yin

Table S2. The clinical characteristics of 155 patients with ovarian cancer

Character	Outcome	N (%)
Age (years) [a, b]	>51	80 (51.95%)
	≤51	74 (48.05%)
Disease-free survival status	Relapse-free	30 (19.35%)
	Relapse	125 (80.65%)
Disease-free survival time (months) [c]	>43	63 (40.65%)
	≤43	92 (59.35%)
Overall survival status	Alive	72 (46.45%)
	Dead	83 (53.55%)
Overall survival time (months) [d]	>58	84 (54.19%)
	≤58	71 (45.81%)
Tumor type	Primary	141 (90.97%)
	Metastasis	14 (9.03%)
Grade [a]	I	15 (11.81%)
	II	8 (6.30%)
	III	104 (81.89%)
TNM staging (AJCC-7)	I	9 (5.81%)
	II	36 (23.23%)
	III	77 (49.68%)
	IV	33 (21.29%)
Pathologic T	T1	9 (5.81%)
	T2	36 (23.23%)
	T3	110 (70.97%)
Pathologic N	N0	114 (73.55%)
	N1	41 (26.45%)
Pathologic M	M0	122 (78.71%)
	M1	33 (21.29%)
Tumor size (cm)	>10	94 (60.65%)
	≤10	61 (39.35%)

Note: N, the number of cases; a, data missing; b, the cut-off point was a mean age of 51 years; c, the cut-off point was a mean disease-free survival of 43 months; d, the cut-off point was a mean overall survival of 43 months; AJCC-7, the 7th edition of the AJCC Cancer Staging Manual.

Table S3. Associations of the top 20 two-gene combinations with overall survival in ovarian cancer patients from Kaplan-Meier plotter, with the smallest *P* value

Two-gene combination *		Overall survival (median)					
		Estimate	Std. error #	95% CI #		*P* value	Difference of estimate #
				Lower	Upper		
EFEMP1 + GATA6	Unfavorable expression (H+H)	32.000	1.904	28.268	35.732	1.28E-12	22.97
	Favorable expression (L+L)	54.970	3.015	49.061	60.879		
	All	44.130	1.764	40.673	47.587		
ALDH1A2 + PSD4	Unfavorable expression (H+L)	35.770	2.906	30.074	41.466	1.13E-11	23.00
	Favorable expression (L+H)	58.770	4.104	50.725	66.815		
	All	45.970	2.262	41.536	50.404		
PLAU + UBL3	Unfavorable expression (H+H)	26.230	3.402	19.561	32.899	3.03E-11	22.07
	Favorable expression (L+L)	48.300	2.090	44.203	52.397		
	All	45.770	1.969	41.911	49.629		
GATA6 + NR2F1	Unfavorable expression (H+H)	34.130	1.617	30.961	37.299	9.54E-11	26.87
	Favorable expression (L+L)	61.000	5.638	49.949	72.051		
	All	45.530	2.212	41.194	49.866		
ALDH1A2 + HSF1	Unfavorable expression (H+L)	35.800	2.439	31.020	40.580	4.16E-10	21.30
	Favorable expression (L+H)	57.100	4.902	47.492	66.708		
	All	45.630	2.020	41.671	49.589		
ECM2 + GATA6	Unfavorable expression (H+H)	29.080	2.313	24.547	33.613	4.74E-10	21.45
	Favorable expression (L+L)	50.530	2.194	46.230	54.830		
	All	46.520	2.015	42.571	50.469		
EFEMP1 + PSD4	Unfavorable expression (H+L)	35.470	2.735	30.109	40.831	5.55E-10	19.50
	Favorable expression (L+H)	54.970	3.512	48.087	61.853		

	All	45.130	2.031	41.148	49.112		
PLAU + PRPF40A	Unfavorable expression (H+H)	29.080	2.503	24.174	33.986	6.70E-10	21.95
	Favorable expression (L+L)	51.030	3.163	44.831	57.229		
	All	45.000	2.362	40.371	49.629		
ALDH1A2 + FAM13A	Unfavorable expression (H+L)	35.000	2.968	29.182	40.818	7.58E-10	25.29
	Favorable expression (L+H)	60.290	4.683	51.111	69.469		
	All	48.000	2.380	43.334	52.666		
MARS + ALDH1A2	Unfavorable expression (H+H)	36.730	1.831	33.141	40.319	7.75E-10	25.77
	Favorable expression (L+L)	62.500	4.019	54.622	70.378		
	All	43.930	2.007	39.996	47.864		
ALDH1A2 + UBL3	Unfavorable expression (H+H)	36.000	2.511	31.078	40.922	1.06E-09	21.87
	Favorable expression (L+L)	57.870	4.575	48.903	66.837		
	All	45.770	2.306	41.251	50.289		
LSM4 + DCN	Unfavorable expression (H+H)	33.770	2.429	29.009	38.531	1.15E-09	21.23
	Favorable expression (L+L)	55.000	4.318	46.537	63.463		
	All	44.770	1.950	40.948	48.592		
ALDH1A2 + TGFBR3	Unfavorable expression (H+H)	36.010	3.397	29.352	42.668	1.46E-09	24.28
	Favorable expression (L+L)	60.290	5.700	49.118	71.462		
	All	48.000	2.936	42.246	53.754		
GATA6 + MYCL1	Unfavorable expression (H+L)	32.000	2.162	27.763	36.237	2.02E-09	20.00
	Favorable expression (L+H)	52.000	3.099	45.926	58.074		
	All	44.530	1.920	40.767	48.293		
NR2F1 + PSD4	Unfavorable expression (H+L)	38.600	2.121	34.443	42.757	1.92E-09	21.50
	Favorable expression (L+H)	60.100	3.457	53.325	66.875		
	All	45.730	1.913	41.980	49.480		

PLAU + GATA6	Unfavorable expression (H+H)	29.400	1.962	25.554	33.246	1.72E-09	23.37
	Favorable expression (L+L)	52.770	2.350	48.164	57.376		
	All	48.550	2.211	44.215	52.885		
ALDH1A2 + DCN	Unfavorable expression (H+H)	36.030	2.591	30.952	41.108	1.47E-09	20.97
	Favorable expression (L+L)	57.000	3.053	51.016	62.984		
	All	46.520	2.025	42.551	50.489		
ALDH1A2 + GATA6	Unfavorable expression (H+H)	33.800	1.750	30.369	37.231	5.47E-11	24.97
	Favorable expression (L+L)	58.770	4.228	50.484	67.056		
	All	45.800	2.566	40.771	50.829		
DCN + GATA6	Unfavorable expression (H+H)	27.730	2.124	23.568	31.892	3.04E-12	22.80
	Favorable expression (L+L)	50.530	2.314	45.994	55.066		
	All	48.000	1.969	44.141	51.859		
GATA6 + PDGFRA	Unfavorable expression (H+H)	32.000	1.681	28.705	35.295	1.56E-09	22.83
	Favorable expression (L+L)	54.830	5.078	44.877	64.783		
	All	40.100	2.151	35.884	44.316		

Note: The gene transcript levels were dichotomized into high and low expression with auto-selected best cutoff by the Kaplan-Meier plotter.

* Samples with high (H) and/or low (L) expression of two genes in the combination were assigned as one group, and samples with the opposite expression of the two genes were assigned as the other group. These two groups were compared with Kaplan-Meier curves to reflect the expression of the combined genes with prognosis. The group with long survival time was named as “favorable expression”, and the group with short survival time was named as “unfavorable expression”.

# Std. error, standard error; 95% CI, 95% confidence interval; Difference of estimate, median estimated survival time of favorable expression - median estimated survival time of unfavorable expression. Statistical tests: Kaplan-Meier survival analysis with log-rank test.

Table S4. Associations of the top 20 two-gene combinations with progression-free survival in ovarian cancer patients from Kaplan-Meier plotter, with the smallest *P* value

Two-gene combination *		Progression-free survival (median)					
		Estimate	Std. error #	95% CI #		*P* value	Difference of estimate #
				Lower	Upper		
PRCC + DCN	Unfavorable expression (H+H)	14.000	0.743	12.543	15.457	3.10E-15	19.43
	Favorable expression (L+L)	33.430	5.625	22.404	44.456		
	All	19.000	1.096	16.853	21.147		
CEBPG + DCN	Unfavorable expression (H+H)	14.670	0.951	12.805	16.535	7.29E-15	24.30
	Favorable expression (L+L)	38.970	5.951	27.307	50.633		
	All	19.800	1.076	17.690	21.910		
PRCC + MEF2C	Unfavorable expression (H+H)	14.530	1.039	12.494	16.566	2.79E-14	18.81
	Favorable expression (L+L)	33.340	4.645	24.235	42.445		
	All	20.000	1.321	17.412	22.588		
ARL4C + MEF2C	Unfavorable expression (H+H)	14.000	0.792	12.448	15.552	5.05E-14	14.00
	Favorable expression (L+L)	28.000	2.034	24.013	31.987		
	All	20.000	0.821	18.391	21.609		
KRIT1 + EFEMP1	Unfavorable expression (H+H)	15.000	0.833	13.367	16.633	1.20E-13	13.23
	Favorable expression (L+L)	28.230	2.144	24.028	32.432		
	All	19.000	0.909	17.217	20.783		
MARS + DCN	Unfavorable expression (H+H)	14.000	0.640	12.746	15.254	1.05E-13	20.55
	Favorable expression (L+L)	34.550	5.459	23.850	45.250		
	All	18.430	1.009	16.452	20.408		
KRIT1 + FHL1	Unfavorable expression (H+H)	16.000	0.997	14.045	17.955	1.64E-13	13.03
	Favorable expression (L+L)	29.030	2.442	24.244	33.816		

	All	20.000	0.878	18.280	21.720		
ARL4C + DCN	Unfavorable expression (H+H)	14.030	0.682	12.693	15.367	4.46E-13	13.17
	Favorable expression (L+L)	27.200	1.903	23.470	30.930		
	All	19.550	0.844	17.895	21.205		
CEBPG + MEF2C	Unfavorable expression (H+H)	15.500	0.946	13.645	17.355	3.25E-13	18.67
	Favorable expression (L+L)	34.170	6.204	22.011	46.329		
	All	20.930	1.205	18.568	23.292		
DCN + FHL1	Unfavorable expression (H+H)	14.530	0.889	12.787	16.273	5.26E-13	13.09
	Favorable expression (L+L)	27.620	2.350	23.013	32.227		
	All	20.070	0.849	18.406	21.734		
FHL1 + MEF2C	Unfavorable expression (H+H)	14.170	0.786	12.630	15.710	7.26E-13	12.83
	Favorable expression (L+L)	27.000	2.315	22.463	31.537		
	All	20.000	0.848	18.339	21.661		
PEA15 + PRKCI	Unfavorable expression (H+H)	17.500	1.013	15.515	19.485	4.34E-13	25.08
	Favorable expression (L+L)	42.580	6.268	30.295	54.865		
	All	21.000	1.027	18.987	23.013		
MARS + MEF2C	Unfavorable expression (H+H)	14.900	1.095	12.753	17.047	7.56E-13	18.63
	Favorable expression (L+L)	33.530	5.363	23.018	44.042		
	All	19.400	0.893	17.649	21.151		
ARL4C + PRKCI	Unfavorable expression (H+H)	15.600	1.069	13.505	17.695	9.00E-13	15.27
	Favorable expression (L+L)	30.870	2.647	25.682	36.058		
	All	21.770	1.110	19.594	23.946		
PRKCI + DCN	Unfavorable expression (H+H)	13.200	0.504	12.212	14.188	1.01E-12	13.08
	Favorable expression (L+L)	26.280	1.443	23.451	29.109		
	All	21.590	1.019	19.592	23.588		

FHL1 + UBB	Unfavorable expression (H+L)	16.000	0.975	14.090	17.910	9.70E-13	15.90
	Favorable expression (L+H)	31.900	5.440	21.238	42.562		
	All	20.000	0.905	18.227	21.773		
PRCC + MAF	Unfavorable expression (H+H)	15.000	1.002	13.036	16.964	1.10E-12	16.27
	Favorable expression (L+L)	31.270	4.710	22.039	40.501		
	All	19.400	1.195	17.058	21.742		
PRKCI + FHL1	Unfavorable expression (H+H)	15.000	0.967	13.105	16.895	1.50E-12	13.35
	Favorable expression (L+L)	28.350	2.526	23.398	33.302		
	All	20.000	0.957	18.125	21.875		
PRKCI + EFEMP1	Unfavorable expression (H+H)	15.000	0.664	13.698	16.302	1.26E-12	18.63
	Favorable expression (L+L)	33.630	3.976	25.836	41.424		
	All	20.770	1.130	18.556	22.984		
ARL4C + KRIT1	Unfavorable expression (H+H)	16.630	0.946	14.775	18.485	1.71E-12	14.03
	Favorable expression (L+L)	30.660	2.159	26.429	34.891		
	All	20.930	1.115	18.744	23.116		

Note: The gene transcript levels were dichotomized into high and low expression with auto-selected best cutoff by the Kaplan-Meier plotter.

* Samples with high (H) and/or low (L) expression of two genes in the combination were assigned as one group, and samples with the opposite expression of the two genes were assigned as the other group. These two groups were compared with Kaplan-Meier curves to reflect the expression of the combined genes with prognosis. The group with long survival time was named as “favorable expression”, and the group with short survival time was named as “unfavorable expression”.

# Std. error, standard error; 95% CI, 95% confidence interval; Difference of estimate, median estimated survival time of favorable expression - median estimated survival time of unfavorable expression. Statistical tests: Kaplan-Meier survival analysis with log-rank test.

Table S5. Associations of the top 20 three-gene combinations with overall survival in ovarian cancer patients from Kaplan-Meier plotter, with the smallest *P* value

Three-gene combination *		Overall survival (median)					
		Estimate	Std. error #	95% CI #		*P* value	Difference of estimate #
				Lower	Upper		
PLAU + ALDH1A2 + UBL3	Unfavorable expression (H+H+H)	28.670	3.197	22.404	34.936	1.46E-15	30.26
	Favorable expression (L+L+L)	58.930	3.934	51.220	66.640		
	All	49.570	3.343	43.018	56.122		
PLAU + NR2F1 + UBL3	Unfavorable expression (H+H+H)	28.670	3.424	21.958	35.382	6.82E-15	36.20
	Favorable expression (L+L+L)	64.870	5.963	53.183	76.557		
	All	52.770	3.824	45.274	60.266		
PLAU + ECM2 + UBL3	Unfavorable expression (H+H+H)	26.180	3.111	20.082	32.278	7.21E-14	22.19
	Favorable expression (L+L+L)	48.370	2.326	43.811	52.929		
	All	45.630	1.997	41.716	49.544		
ECM2 + ALDH1A2 + GATA6	Unfavorable expression (H+H+H)	28.670	2.248	24.265	33.075	1.25E-13	30.26
	Favorable expression (L+L+L)	58.930	4.807	49.509	68.351		
	All	48.300	3.498	41.444	55.156		
DCN + GATA6 + NR2F1	Unfavorable expression (H+H+H)	28.000	2.076	23.931	32.069	1.02E-13	36.97
	Favorable expression (L+L+L)	64.970	5.868	53.469	76.471		
	All	48.060	3.699	40.809	55.311		
ALDH1A2 + UBB + HSF1	Unfavorable expression (H+L+L)	32.300	2.749	26.912	37.688	8.11E-14	32.70
	Favorable expression (L+H+H)	65.000	6.050	53.141	76.859		
	All	49.470	2.818	43.948	54.992		
LSM4 + PLAU + AKT3	Unfavorable expression (H+H+H)	28.000	2.209	23.670	32.330	1.81E-13	29.00
	Favorable expression (L+L+L)	57.000	8.180	40.967	73.033		

	All	43.930	2.740	38.560	49.300		
GATA6 + NR2F1 + PTGIS	Unfavorable expression (H+H+H)	30.000	2.005	26.071	33.929	3.60E-13	31.53
	Favorable expression (L+L+L)	61.530	5.721	50.316	72.744		
	All	48.060	2.460	43.239	52.881		
CKS1B + ALDH1A2 + GATA6	Unfavorable expression (H+H+H)	31.500	1.844	27.887	35.113	3.36E-13	41.67
	Favorable expression (L+L+L)	73.170	7.378	58.709	87.631		
	All	45.630	4.415	36.976	54.284		
DCN + GATA6 + MEF2C	Unfavorable expression (H+H+H)	22.970	2.030	18.990	26.950	3.33E-13	28.00
	Favorable expression (L+L+L)	50.970	2.771	45.539	56.401		
	All	47.820	2.452	43.014	52.626		
ALDH1A2 + EFEMP1 + GATA6	Unfavorable expression (H+H+H)	31.370	1.786	27.870	34.870	7.37E-13	26.50
	Favorable expression (L+L+L)	57.870	5.173	47.730	68.010		
	All	43.000	2.675	37.757	48.243		
ARL4C + ALDH1A2 + CIRBP	Unfavorable expression (H+H+L)	31.700	1.995	27.789	35.611	3.04E-13	36.30
	Favorable expression (L+L+H)	68.000	12.630	43.244	92.756		
	All	48.200	3.425	41.487	54.913		
PLAU + PDGFRA + UBL3	Unfavorable expression (H+H+H)	26.180	3.227	19.854	32.506	4.98E-13	28.82
	Favorable expression (L+L+L)	55.000	4.238	46.693	63.307		
	All	45.170	3.062	39.168	51.172		
ESPL1 + DCN + GATA6	Unfavorable expression (H+H+H)	25.000	2.199	20.690	29.310	3.05E-13	37.50
	Favorable expression (L+L+L)	62.500	6.790	49.192	75.808		
	All	46.520	5.099	36.526	56.514		
LSM4 + PLAU + GATA6	Unfavorable expression (H+H+H)	28.670	2.816	23.150	34.190	1.49E-13	36.33
	Favorable expression (L+L+L)	65.000	7.132	51.022	78.978		
	All	55.000	4.686	45.815	64.185		

EFEMP1 + GATA6 + NR2F1	Unfavorable expression (H+H+H)	30.540	1.874	26.867	34.213	8.87E-13	28.23
	Favorable expression (L+L+L)	58.770	6.193	46.632	70.908		
	All	40.430	2.456	35.616	45.244		
DCN + NR2F1 + TGFBR3	Unfavorable expression (H+H+H)	28.570	3.400	21.907	35.233	5.25E-13	32.96
	Favorable expression (L+L+L)	61.530	6.164	49.449	73.611		
	All	48.300	3.120	42.184	54.416		
DCN + EFEMP1 + GATA6	Unfavorable expression (H+H+H)	27.730	2.005	23.799	31.661	8.77E-13	27.10
	Favorable expression (L+L+L)	54.830	3.053	48.846	60.814		
	All	46.520	3.071	40.501	52.539		
MRPS12 + PRPF40A + PTGIS	Unfavorable expression (H+H+H)	24.500	4.477	15.725	33.275	1.27E-13	33.50
	Favorable expression (L+L+L)	58.000	7.188	43.912	72.088		
	All	50.970	3.641	43.834	58.106		
ALDH1A2 + DCN + GATA6	Unfavorable expression (H+H+H)	28.670	2.048	24.657	32.683	6.52E-15	31.30
	Favorable expression (L+L+L)	59.970	3.592	52.929	67.011		
	All	49.430	3.197	43.164	55.696		

Note: The gene transcript levels were dichotomized into high and low expression with auto-selected best cutoff by the Kaplan-Meier plotter.

* Samples with high (H) and/or low (L) expression of three genes in the combination were assigned as one group, and samples with the opposite expression of the three genes were assigned as the other group. These two groups were compared with Kaplan-Meier curves to reflect the expression of the combined genes with prognosis. The group with long survival time was named as “favorable expression”, and the group with short survival time was named as “unfavorable expression”.

# Std. error, standard error; 95% CI, 95% confidence interval; Difference of estimate, median estimated survival time of favorable expression - median estimated survival time of unfavorable expression. Statistical tests: Kaplan-Meier survival analysis with log-rank test.

Table S6. Associations of the top 20 three-gene combinations with progression-free survival in ovarian cancer patients from Kaplan-Meier plotter, with the smallest *P* value

Three-gene combination *		Progression-free survival (median)					
		Estimate	Std. error #	95% CI #		*P* value	Difference of estimate #
				Lower	Upper		
KRIT1 + MARS + MEF2C	Unfavorable expression (H+H+H)	14.000	1.148	11.749	16.251	1.04E-18	30.29
	Favorable expression (L+L+L)	44.290	5.599	33.316	55.264		
	All	21.000	1.693	17.682	24.318		
PRCC + FHL1 + MEF2C	Unfavorable expression (H+H+H)	13.000	0.759	11.513	14.487	2.79E-18	34.61
	Favorable expression (L+L+L)	47.610	8.375	31.195	64.025		
	All	18.300	1.216	15.917	20.683		
CP + FHL1 + MEF2C	Unfavorable expression (H+H+H)	14.000	0.656	12.715	15.285	4.20E-18	26.70
	Favorable expression (L+L+L)	40.700	6.039	28.864	52.536		
	All	21.000	1.761	17.548	24.452		
PRCC + DCN + MEF2C	Unfavorable expression (H+H+H)	12.670	0.731	11.237	14.103	4.96E-18	29.06
	Favorable expression (L+L+L)	41.730	6.151	29.674	53.786		
	All	20.000	1.757	16.557	23.443		
CEBPG + KRIT1 + MEF2C	Unfavorable expression (H+H+H)	14.170	0.991	12.227	16.113	5.98E-18	30.85
	Favorable expression (L+L+L)	45.020	8.998	27.383	62.657		
	All	23.000	2.252	18.587	27.413		
CKS1B + FHL1 + MEF2C	Unfavorable expression (H+H+H)	13.000	0.937	11.163	14.837	1.02E-17	30.19
	Favorable expression (L+L+L)	43.190	6.836	29.791	56.589		
	All	18.230	1.321	15.642	20.818		
CEBPG + DCN + GATA6	Unfavorable expression (H+H+H)	14.000	1.012	12.017	15.983	1.04E-17	33.94
	Favorable expression (L+L+L)	47.940	12.237	23.955	71.925		

	All	23.570	2.495	18.680	28.460		
ARL4C + KRIT1 + UBB	Unfavorable expression (H+H+L)	13.730	0.923	11.921	15.539	4.44E-17	29.46
	Favorable expression (L+L+H)	43.190	5.435	32.537	53.843		
	All	21.290	1.665	18.026	24.554		
KRIT1 + PRCC + MEF2C	Unfavorable expression (H+H+H)	13.000	1.083	10.878	15.122	3.73E-17	29.58
	Favorable expression (L+L+L)	42.580	6.774	29.303	55.857		
	All	23.360	2.226	18.998	27.722		
CEBPG + PRKCI + DCN	Unfavorable expression (H+H+H)	13.200	0.563	12.097	14.303	1.64E-17	42.16
	Favorable expression (L+L+L)	55.360	7.071	41.500	69.220		
	All	22.600	2.035	18.612	26.588		
CEBPG + DCN + UBB	Unfavorable expression (H+H+L)	12.230	0.694	10.869	13.591	6.86E-18	56.88
	Favorable expression (L+L+H)	69.110	11.936	45.716	92.504		
	All	21.000	2.101	16.882	25.118		
PRKCI + DCN + MAF	Unfavorable expression (H+H+H)	12.000	0.561	10.900	13.100	1.39E-16	15.00
	Favorable expression (L+L+L)	27.000	2.056	22.970	31.030		
	All	22.000	1.347	19.360	24.640		
YWHAZ + FHL1 + UBB	Unfavorable expression (H+H+L)	14.530	0.853	12.859	16.201	3.20E-17	32.07
	Favorable expression (L+L+H)	46.600	5.707	35.415	57.785		
	All	21.130	1.247	18.687	23.573		
KRIT1 + CP + FHL1	Unfavorable expression (H+H+H)	15.100	1.045	13.051	17.149	1.23E-16	28.09
	Favorable expression (L+L+L)	43.190	6.392	30.661	55.719		
	All	19.980	1.318	17.396	22.564		
FHL1 + MEF2C + UBB	Unfavorable expression (H+H+L)	11.920	1.313	9.347	14.493	7.55E-17	21.08
	Favorable expression (L+L+H)	33.000	6.425	20.407	45.593		
	All	21.930	1.608	18.778	25.082		

CEBPG + PRCC + DCN	Unfavorable expression (H+H+H)	14.000	0.845	12.344	15.656	3.70E-17	54.50
	Favorable expression (L+L+L)	68.500	8.117	52.591	84.409		
	All	19.000	1.484	16.090	21.910		
MARS + DCN + MEF2C	Unfavorable expression (H+H+H)	13.000	0.819	11.395	14.605	2.30E-16	31.29
	Favorable expression (L+L+L)	44.290	5.989	32.552	56.028		
	All	19.000	1.320	16.413	21.587		
PRKCI + NR2F1 + UBB	Unfavorable expression (H+H+L)	12.230	0.694	10.870	13.590	2.03E-16	20.84
	Favorable expression (L+L+H)	33.070	5.904	21.498	44.642		
	All	19.000	1.179	16.689	21.311		
KRIT1 + DCN + FHL1	Unfavorable expression (H+H+H)	13.130	0.794	11.574	14.686	3.75E-16	20.40
	Favorable expression (L+L+L)	33.530	3.560	26.553	40.507		
	All	21.930	1.753	18.493	25.367		
CEBPG + KRIT1 + DCNN	Unfavorable expression (H+H+H)	13.600	1.013	11.614	15.586	2.02E-16	34.95
	Favorable expression (L+L+L)	48.550	11.339	26.325	70.775		
	All	21.930	2.135	17.745	26.115		

Note: The gene transcript levels were dichotomized into high and low expression with auto-selected best cutoff by the Kaplan-Meier plotter.

* Samples with high (H) and/or low (L) expression of three genes in the combination were assigned as one group, and samples with the opposite expression of the three genes were assigned as the other group. These two groups were compared with Kaplan-Meier curves to reflect the expression of the combined genes with prognosis. The group with long survival time was named as “favorable expression”, and the group with short survival time was named as “unfavorable expression”.

# Std. error, standard error; 95% CI, 95% confidence interval; Difference of estimate, median estimated survival time of favorable expression - median estimated survival time of unfavorable expression. Statistical tests: Kaplan-Meier survival analysis with log-rank test.

Table S7. Associations of the top 20 four-gene combinations with overall survival in ovarian cancer patients from Kaplan-Meier plotter, with the smallest *P* value

Four-gene combination *		Overall survival (median)					
		Estimate	Std. error #	95% CI #		*P* value	Difference of estimate #
				Lower	Upper		
DDX39 + ALDH1A2 + GATA6 + MAOB	Unfavorable expression (H+H+H+L)	26.200	5.517	15.387	37.013	1.02E-17	51.13
	Favorable expression (L+L+L+H)	77.330	6.584	64.426	90.234		
	All	61.570	5.342	51.100	72.040		
CEBPG + MRPS12 + PTGIS + MAOB	Unfavorable expression (H+H+H+L)	19.020	4.601	10.001	28.039	1.37E-16	37.98
	Favorable expression (L+L+L+H)	57.000	2.888	51.339	62.661		
	All	46.520	3.483	39.692	53.348		
PEA15 + GATA6 + AMIGO2 + UBB	Unfavorable expression (H+H+L+L)	34.130	1.412	31.363	36.897	4.72E-17	60.87
	Favorable expression (L+L+H+H)	95.000	7.248	80.794	109.206		
	All	54.670	3.832	47.160	62.180		
EFEMP1 + NR2F1 + TGFBR3 + HSF1	Unfavorable expression (H+H+H+L)	30.630	5.221	20.397	40.863	1.38E-15	34.34
	Favorable expression (L+L+L+H)	64.970	6.457	52.313	77.627		
	All	45.970	3.239	39.621	52.319		
CEBPG + MRPS12 + PTGIS + ASMTL	Unfavorable expression (H+H+H+L)	15.900	1.464	13.030	18.770	2.40E-16	44.07
	Favorable expression (L+L+L+H)	59.970	6.997	46.256	73.684		
	All	53.330	4.711	44.096	62.564		
MRPS12 + VEGFA + PTGIS + METTL7A	Unfavorable expression (H+H+H+L)	17.000	3.871	9.412	24.588	7.30E-17	78.00
	Favorable expression (L+L+L+H)	95.000	14.574	66.436	123.564		
	All	50.530	9.288	32.326	68.734		
MRPS12 + ALDH1A2 + MAOB + BOP1	Unfavorable expression (H+H+L+L)	17.170	2.695	11.887	22.453	7.04E-18	57.83
	Favorable expression (L+L+H+H)	75.000	11.474	52.510	97.490		

	All	54.830	8.142	38.871	70.789		
PLAU + SFRP1 + AKT3 + ASMTL	Unfavorable expression (H+H+H+L)	16.000	0.654	14.718	17.282	1.00E-17	41.00
	Favorable expression (L+L+L+H)	57.000	4.400	48.377	65.623		
	All	53.270	4.199	45.040	61.500		
DCN + NR2F1 + TGFBR3 + FLAD1	Unfavorable expression (H+H+H+L)	25.890	2.911	20.184	31.596	5.41E-15	47.11
	Favorable expression (L+L+L+H)	73.000	8.407	56.522	89.478		
	All	48.300	4.253	39.965	56.635		
ARL4C + ALDH1A2 + GATA6 + CIRBP	Unfavorable expression (H+H+H+L)	29.030	4.202	20.795	37.265	2.71E-15	62.50
	Favorable expression (L+L+L+H)	91.530	13.407	65.252	117.808		
	All	57.000	4.587	48.009	65.991		
PLAU + SFRP1 + EFEMP1 + KIF22	Unfavorable expression (H+H+H+L)	18.930	1.853	15.298	22.562	1.02E-14	37.89
	Favorable expression (L+L+L+H)	56.820	4.555	47.892	65.748		
	All	50.370	3.983	42.563	58.177		
DDX39 + MRPS12 + EFEMP1 + ASMTL	Unfavorable expression (H+H+H+L)	18.730	1.505	15.780	21.680	7.36E-15	40.17
	Favorable expression (L+L+L+H)	58.900	6.508	46.145	71.655		
	All	53.270	4.469	44.511	62.029		
CAV1 + NR2F1 + FLAD1 + PSD4	Unfavorable expression (H+H+L+L)	32.300	2.466	27.467	37.133	6.51E-14	39.48
	Favorable expression (L+L+H+H)	71.780	7.188	57.692	85.868		
	All	47.800	2.317	43.259	52.341		
CAV1 + GATA6 + NR2F1 + MYCL1	Unfavorable expression (H+H+H+L)	31.400	2.291	26.910	35.890	5.35E-14	41.77
	Favorable expression (L+L+L+H)	73.170	7.946	57.596	88.744		
	All	46.600	3.635	39.475	53.725		
MRPS12 + PTGIS + ASMTL + MYCL1	Unfavorable expression (H+H+L+L)	17.000	1.567	13.929	20.071	2.49E-15	38.93
	Favorable expression (L+L+H+H)	55.930	9.289	37.724	74.136		
	All	50.000	5.277	39.658	60.342		

PLAU + SFRP1 + NR2F1 + KIF22	Unfavorable expression (H+H+H+L)	21.000	1.481	18.097	23.903	3.95E-15	43.97
	Favorable expression (L+L+L+H)	64.970	6.912	51.422	78.518		
	All	53.270	5.769	41.963	64.577		
ARL4C + ALDH1A2 + CIRBP + WDR19	Unfavorable expression (H+H+L+L)	32.070	4.204	23.831	40.309	9.91E-15	59.46
	Favorable expression (L+L+H+H)	91.530	18.884	54.517	128.543		
	All	55.000	3.854	47.446	62.554		
PLAU + SFRP1 + FLAD1 + KIF22	Unfavorable expression (H+H+L+L)	18.930	1.758	15.485	22.375	1.21E-14	34.34
	Favorable expression (L+L+H+H)	53.270	4.974	43.521	63.019		
	All	48.000	3.539	41.064	54.936		
MRPS12 + PTGIS + ASMTL + METTL7A	Unfavorable expression (H+H+L+L)	14.000	2.014	10.053	17.947	7.90E-16	43.00
	Favorable expression (L+L+H+H)	57.000	7.635	42.036	71.964		
	All	55.370	4.898	45.770	64.970		
ALDH1A2 + DCN + GATA6 + PDGFRA	Unfavorable expression (H+H+H+H)	28.000	1.797	24.479	31.521	1.82E-09	29.00
	Favorable expression (L+L+L+L)	57.000	9.929	37.539	76.461		
	All	37.960	3.547	31.009	44.911		

Note: The gene transcript levels were dichotomized into high and low expression with auto-selected best cutoff by the Kaplan-Meier plotter

* Samples with high (H) and/or low (L) expression of four genes in the combination were assigned as one group, and samples with the opposite expression of the four genes were assigned as the other group. These two groups were compared with Kaplan-Meier curves to reflect the expression of the combined genes with prognosis. The group with long survival time was named as “favorable expression”, and the group with short survival time was named as “unfavorable expression”.

# Std. error, standard error; 95% CI, 95% confidence interval; Difference of estimate, median estimated survival time of favorable expression - median estimated survival time of unfavorable expression. Statistical tests: Kaplan-Meier survival analysis with log-rank test.

Table S8. Associations of the top 20 four-gene combinations with progression-free survival in ovarian cancer patients from Kaplan-Meier plotter, with the smallest *P* value

Four-gene combination *		Progression-free survival (median)					
		Estimate	Std. error #	95% CI #		*P* value	Difference of estimate #
				Lower	Upper		
CKS1B + DCN + UFSP2 + HSF1	Unfavorable expression (H+H+H+L)	11.000	0.395	10.227	11.773	3.00E-18	31.58
	Favorable expression (L+L+L+H)	42.580	6.898	29.060	56.100		
	All	19.400	2.057	15.368	23.432		
CEBPG + DCN + GATA6 + UBB	Unfavorable expression (H+H+H+L)	10.930	1.549	7.894	13.966	7.79E-21	68.64
	Favorable expression (L+L+L+H)	79.570	8.927	62.074	97.066		
	All	30.000	6.464	17.331	42.669		
ARL4C + KRIT1 + YWHAZ + UBB	Unfavorable expression (H+H+H+L)	13.430	1.025	11.422	15.438	5.39E-18	34.63
	Favorable expression (L+L+L+H)	48.060	7.652	33.063	63.057		
	All	22.390	2.049	18.373	26.407		
CKS1B + DCN + HSF1 + MYCL1	Unfavorable expression (H+H+L+L)	11.000	0.607	9.811	12.189	1.58E-18	19.78
	Favorable expression (L+L+H+H)	30.780	4.741	21.487	40.073		
	All	21.550	2.614	16.427	26.673		
CKS1B + AKT3 + DCN + HSF1	Unfavorable expression (H+H+H+L)	11.100	0.593	9.938	12.262	5.13E-18	27.59
	Favorable expression (L+L+L+H)	38.690	7.176	24.625	52.755		
	All	19.400	1.799	15.874	22.926		
KRIT1 + YWHAZ + DCN + UBB	Unfavorable expression (H+H+H+L)	8.230	1.551	5.190	11.270	8.92E-19	24.14
	Favorable expression (L+L+L+H)	32.370	7.564	17.545	47.195		
	All	24.000	2.139	19.808	28.192		
ARL4C + GATA6 + MAF + HSF1	Unfavorable expression (H+H+H+L)	11.000	1.211	8.626	13.374	1.64E-18	20.27
	Favorable expression (L+L+L+H)	31.270	4.924	21.619	40.921		

	All	26.520	2.687	21.253	31.787		
ARL4C + DCN + UFSP2 + HSF1	Unfavorable expression (H+H+H+L)	11.400	0.476	10.467	12.333	3.15E-17	31.18
	Favorable expression (L+L+L+H)	42.580	6.367	30.100	55.060		
	All	21.000	2.437	16.224	25.776		
ARL4C + DCN + GATA6 + MYCL1	Unfavorable expression (H+H+H+L)	13.170	1.614	10.007	16.333	8.31E-18	30.02
	Favorable expression (L+L+L+H)	43.190	6.441	30.565	55.815		
	All	27.960	3.058	21.967	33.953		
FHL1 + NR2F1 + HSF1 + MYCL1	Unfavorable expression (H+H+L+L)	13.000	0.720	11.588	14.412	1.33E-17	35.55
	Favorable expression (L+L+H+H)	48.550	6.427	35.953	61.147		
	All	21.000	2.232	16.625	25.375		
ARL4C + DCN + HSF1 + MYCL1	Unfavorable expression (H+H+L+L)	12.000	0.831	10.372	13.628	3.46E-17	25.00
	Favorable expression (L+L+H+H)	37.000	4.964	27.271	46.729		
	All	24.700	2.656	19.494	29.906		
MEF2C + NR2F1 + NBR1+ HSF1	Unfavorable expression (H+H+H+L)	14.000	0.929	12.178	15.822	4.69E-17	38.57
	Favorable expression (L+L+L+H)	52.570	7.048	38.755	66.385		
	All	23.360	2.635	18.195	28.525		
CP + FHL1 + MEF2C + UBB	Unfavorable expression (H+H+H+L)	11.000	1.028	8.986	13.014	4.58E-18	37.55
	Favorable expression (L+L+L+H)	48.550	6.744	35.333	61.767		
	All	22.410	2.467	17.574	27.246		
CEBPG + DCN + MEF2C + UBB	Unfavorable expression (H+H+H+L)	11.920	1.532	8.917	14.923	4.69E-18	64.73
	Favorable expression (L+L+L+H)	76.650	8.515	59.961	93.339		
	All	25.530	3.425	18.816	32.244		
CEBPG + GATA6 +MAF + UBB	Unfavorable expression (H+H+H+L)	13.170	1.162	10.893	15.447	3.42E-18	52.65
	Favorable expression (L+L+L+H)	65.820	11.209	43.851	87.789		
	All	28.230	2.410	23.507	32.953		

ARL4C + FHL1 + HSF1 + MYCL1	Unfavorable expression (H+H+L+L)	13.170	0.940	11.328	15.012	7.73E-17	35.13
	Favorable expression (L+L+H+H)	48.300	9.214	30.240	66.360		
	All	20.470	2.366	15.833	25.107		
DCN + FHL1 + MEF2C + UBB	Unfavorable expression (H+H+H+L)	10.000	1.523	7.016	12.984	3.44E-17	30.70
	Favorable expression (L+L+L+H)	40.700	7.484	26.031	55.369		
	All	22.570	2.034	18.584	26.556		
ARL4C + KRIT1 + UBL3 + UBB	Unfavorable expression (H+H+H+L)	13.000	0.911	11.215	14.785	9.75E-17	42.97
	Favorable expression (L+L+L+H)	55.970	9.856	36.652	75.288		
	All	21.130	2.108	16.998	25.262		
EIF4G1 + AKT3 + MEF2C + HSF1	Unfavorable expression (H+H+H+L)	11.000	0.546	9.929	12.071	5.00E-17	23.17
	Favorable expression (L+L+L+H)	34.170	5.822	22.759	45.581		
	All	22.400	2.851	16.811	27.989		
FHL1 + UFSP2 + HSF1 + MYCL1	Unfavorable expression (H+H+L+L)	12.000	1.058	9.926	14.074	1.84E-16	35.94
	Favorable expression (L+L+H+H)	47.940	7.044	34.134	61.746		
	All	22.410	2.661	17.195	27.625		

Note: The gene transcript levels were dichotomized into high and low expression with auto-selected best cutoff by the Kaplan-Meier plotter.

* Samples with high (H) and/or low (L) expression of four genes in the combination were assigned as one group, and samples with the opposite expression of the four genes were assigned as the other group. These two groups were compared with Kaplan-Meier curves to reflect the expression of the combined genes with prognosis. The group with long survival time was named as "favorable expression", and the group with short survival time was named as "unfavorable expression".

# Std. error, standard error; 95% CI, 95% confidence interval; Difference of estimate, median estimated survival time of favorable expression - median estimated survival time of unfavorable expression. Statistical tests: Kaplan-Meier survival analysis with log-rank test.

Table S9. Associations of ALDH1A2, DCN, GATA6, and PDGFRA independently and in combinations with overall survival, using the immunohistochemistry data of 155 ovarian cancer samples with Kaplan-Meier curves

Gene*	Expression*	Overall survival (mean)					Overall survival (median)					*P* value
		Estimate	Std. error#	95% CI# Lower	95% CI# Upper	Difference of estimate#	Estimate	Std. error#	95% CI# Lower	95% CI# Upper	Difference of estimate#	
ALDH1A2	Unfavorable expression (H)	62.450	4.145	54.327	70.574		52.000	10.288	31.836	72.164		
	Favorable expression (L)	76.332	4.882	66.763	85.900	13.881	—	—	—	—	—	0.028
	All	68.232	3.249	61.863	74.600		76.000	11.900	52.675	99.325		
DCN	Unfavorable expression (H)	58.414	5.125	48.369	68.460		45.000	7.100	31.083	58.917		
	Favorable expression (L)	73.873	4.023	65.988	81.759	15.459	—	—	—	—	—	0.017
	All	68.232	3.249	61.863	74.600		76.000	11.900	52.675	99.325		
GATA6	Unfavorable expression (H)	63.104	3.843	55.572	70.637		52.000	8.855	34.644	69.356		
	Favorable expression (L)	80.427	5.563	69.523	91.331	17.323	—	—	—	—	—	0.016
	All	68.232	3.249	61.863	74.600		76.000	11.900	52.675	99.325		
PDGFRA	Unfavorable expression (H)	56.256	5.116	46.229	66.283		41.000	9.166	23.034	58.966		
	Favorable expression (L)	74.521	3.980	66.720	82.322	18.265	—	—	—	—	—	0.002
	All	68.232	3.249	61.863	74.600		76.000	11.900	52.675	99.325		
ALDH1A2 + DCN	Unfavorable expression (H + H)	56.676	7.070	42.819	70.534		40.000	10.933	18.571	61.429		
	Favorable expression (L + L)	89.488	5.681	78.353	100.622	32.811	—	—	—	—	—	0.001
	All	73.600	5.049	63.704	83.496		—	—	—	—		
ALDH1A2 +	Unfavorable expression (H + H)	59.875	4.306	51.436	68.314		45.000	8.810	27.733	62.267		
GATA6	Favorable expression (L + L)	78.640	6.120	66.645	90.635	18.765	—	—	—	—	—	0.019
	All	65.624	3.658	58.454	72.793		62.000	13.471	35.597	88.403		
DCN + GATA6	Unfavorable expression (H + H)	55.095	6.268	42.810	67.381		40.000	4.861	30.473	49.527		
	Favorable expression (L + L)	90.148	6.738	76.941	103.354	35.053	—	—	—	—	—	0.001
	All	68.762	5.122	58.723	78.801		78.000	—	—	—		
GATA6 + PDGFRA	Unfavorable expression (H + H)	55.245	5.116	45.217	65.273	24.453	40.000	9.978	20.444	59.556	—	0.001
	Favorable expression (L + L)	79.699	5.659	68.607	90.791		—	—	—	—		

	All	66.244	4.019	58.366	74.121		64.000	13.080	38.364	89.636		
ALDH1A2 +	Unfavorable expression (H + H)	53.628	5.206	43.424	63.832		40.000	8.670	23.007	56.993		
PDGFRA	Favorable expression (L + L)	76.134	5.076	66.184	86.084	22.506	—	—	—	—	—	0.001
	All	65.722	3.864	58.148	73.295		62.000	12.096	38.291	85.709		
DCN + PDGFRA	Unfavorable expression (H + H)	59.263	9.292	41.051	77.475		51.000	12.333	26.827	75.173		
	Favorable expression (L + L)	86.375	4.660	77.242	95.509	27.112	—	—	—	—	—	0.004
	All	80.063	4.454	71.334	88.792		—	—	—	—		
ALDH1A2 + DCN +	Unfavorable expression (H + H + H)	56.419	7.448	41.822	71.017		40.000	8.904	22.549	57.451		
GATA6	Favorable expression (L + L + L)	88.028	7.716	72.904	103.152	31.609	—	—	—	—	—	0.011
	All	68.999	5.959	57.319	80.679		78.000	—	—	—		
ALDH1A2 + DCN +	Unfavorable expression (H + H + H)	58.235	9.779	39.069	77.402		51.000	11.662	28.143	73.857		
PDGFRA	Favorable expression (L + L + L)	90.377	5.762	79.084	101.671	32.142	—	—	—	—	—	0.004
	All	79.455	5.718	68.247	90.662		—	—	—	—		
ALDH1A2 +	Unfavorable expression (H + H + H)	53.628	5.206	43.424	63.832		40.000	8.670	23.007	56.993		
GATA6 + PDGFRA	Favorable expression (L + L + L)	75.647	5.912	64.061	87.234	22.019	87.000	—	—	—	47.000	0.003
	All	63.553	4.266	55.193	71.914		58.000	13.702	31.145	84.855		
DCN + GATA6 +	Unfavorable expression (H + H + H)	59.263	9.292	41.051	77.475		51.000	12.333	26.827	75.173		
PDGFRA	Favorable expression (L + L + L)	89.427	6.970	75.765	103.088	30.164	—	—	—	—	—	0.009
	All	76.787	6.123	64.786	88.787		—	—	—	—		
ALDH1A2 + DCN +	Unfavorable expression (H + H + H + H)	58.235	9.779	39.069	77.402		51.000	11.662	28.143	73.857		
GATA6 + PDGFRA	Favorable expression (L + L + L + L)	84.102	7.675	69.059	99.145	25.867	—	—	—	—	—	0.029
	All	73.729	6.867	60.269	87.189		—	—	—	—		

Note: Immunohistochemical scores <5 and ≥5 were defined as low and high gene expression, respectively.

* Samples with high (H) expression of 1, 2, 3, and 4 genes were considered as one group, and samples with low (L) expression of 1, 2, 3, and 4 genes were considered as another group. These two groups were compared with Kaplan-Meier curves to reflect the expression of a single gene or the combined genes with prognosis. The group with long survival time was named as “favorable expression”, and the group with short survival time was named as “unfavorable expression”.

# Std. error, standard error; 95% CI, 95% confidence interval; Difference of estimate, mean or median estimated survival time of favorable expression – mean or median estimated survival time of unfavorable expression. Statistical tests: Kaplan-Meier survival analysis with log-rank test.

Table S10. Associations of ALDH1A2, DCN, GATA6, and PDGFRA independently and in combinations with disease-free survival, using the immunohistochemistry data of 155 ovarian cancer samples with Kaplan-Meier curves

Gene*	Expression*	Disease-free survival (mean)					Disease-free survival (median)					*P* value
		Estimate	Std. error#	95% CI# Lower	95% CI# Upper	Difference of estimate#	Estimate	Std. error#	95% CI# Lower	95% CI# Upper	Difference of estimate#	
ALDH1A2	Unfavorable expression (H)	40.759	3.327	34.238	47.279		29.000	2.939	23.239	34.761		
	Favorable expression (L)	53.876	5.077	43.926	63.826	13.117	35.000	9.120	17.125	52.875	6.000	0.012
	All	45.777	2.887	40.120	51.435		33.000	3.662	25.823	40.177		
DCN	Unfavorable expression (H)	42.013	4.542	33.111	50.915		32.000	4.518	23.144	40.856		
	Favorable expression (L)	47.905	3.691	40.670	55.140	5.892	39.000	6.645	25.975	52.025	7.000	0.356
	All	45.777	2.887	40.120	51.435		33.000	3.662	25.823	40.177		
GATA6	Unfavorable expression (H)	43.786	3.401	37.120	50.451		32.000	4.457	23.264	40.736		
	Favorable expression (L)	50.957	5.324	40.521	61.392	7.171	35.000	8.835	17.683	52.317	3.000	0.225
	All	45.777	2.887	40.120	51.435		33.000	3.662	25.823	40.177		
PDGFRA	Unfavorable expression (H)	39.663	4.627	30.595	48.732		27.000	4.718	17.753	36.247		
	Favorable expression (L)	49.217	3.592	42.177	56.258	9.554	35.000	4.455	26.269	43.731	8.000	0.084
	All	45.777	2.887	40.120	51.435		33.000	3.662	25.823	40.177		

Note: Immunohistochemical scores <5 and ≥5 were defined as low and high gene expression, respectively.

* Samples with high gene expression were considered as one group, and samples with low gene expression were considered as another group. Kaplan-Meier curve was used to compare the relationship between gene expression and prognosis in the two groups. The group with long survival time was named as “favorable expression”, and the group with short survival time was named as “unfavorable expression”.

# Std. error, standard error; 95% CI, 95% confidence interval; Difference of estimate, mean or median estimated survival time of favorable expression – mean or median estimated survival time of unfavorable expression. Statistical tests: Kaplan-Meier survival analysis with log-rank test.

Table S11. Consistency statistics of protein level assessment of ALDH1A2, DCN, GATA6, and PDGFRA in 155 ovarian cancer tissues by three pathologists

Protein	Pathologists	Fleiss' Kappa	Cohen's Kappa	*P* value
ALDH1A2	① vs. ② vs. ③	0.797	—	<0.001
	① vs. ②	—	0.797	<0.001
	① vs. ③	—	0.825	<0.001
	② vs. ③	—	0.769	<0.001
DCN	① vs. ② vs. ③	0.805	—	<0.001
	① vs. ②	—	0.906	<0.001
	① vs. ③	—	0.789	<0.001
	② vs. ③	—	0.724	<0.001
GATA6	① vs. ② vs. ③	0.933	—	<0.001
	① vs. ②	—	0.953	<0.001
	① vs. ③	—	0.935	<0.001
	② vs. ③	—	0.911	<0.001
PDGFRA	① vs. ② vs. ③	0.954	—	<0.001
	① vs. ②	—	0.977	<0.001
	① vs. ③	—	0.949	<0.001
	② vs. ③	—	0.937	<0.001

Note: Statistical methods are Fleiss' Kappa and Cohen's Kappa, which used to assess agreement among all three evaluators and between each pair of evaluators, respectively.

Table S12. Associations of ALDH1A2, DCN, GATA6, and PDGFRA independently and in combinations with overall survival in 107 ovarian cancer patients from the GSE26193 dataset, with Kaplan-Meier curves

Gene*	Expression*	Overall survival (mean)					Overall survival (median)					*P* value
		Estimate	Std. error#	95% CI# Lower	95% CI# Upper	Difference of estimate#	Estimate	Std. error#	95% CI# Lower	95% CI# Upper	Difference of estimate#	
ALDH1A2	Unfavorable expression (H)	52.791	7.262	38.558	67.024		29.080	3.913	21.411	36.749		
	Favorable expression (L)	98.341	13.270	72.331	124.351	45.550	45.630	11.580	22.934	68.326	16.550	0.079
	All	84.338	9.615	65.492	103.184		36.570	5.674	25.448	47.692		
DCN	Unfavorable expression (H)	34.883	5.237	24.619	45.147		27.730	2.030	23.751	31.709		
	Favorable expression (L)	98.691	11.726	75.707	121.674	63.808	46.520	8.514	29.833	63.207	18.790	0.002
	All	84.338	9.615	65.492	103.184		36.570	5.674	25.448	47.692		
GATA6	Unfavorable expression (H)	50.674	5.552	39.793	61.555		34.000	3.643	26.861	41.139		
	Favorable expression (L)	117.911	16.353	85.858	149.963	67.236	60.290	24.585	12.103	108.477	26.290	0.021
	All	84.338	9.615	65.492	103.184		36.570	5.674	25.448	47.692		
PDGFRA	Unfavorable expression (H)	49.995	6.977	36.321	63.670		29.540	2.158	25.311	33.769		
	Favorable expression (L)	100.558	13.841	73.429	127.688	50.563	47.800	6.051	35.940	59.660	18.260	0.025
	All	84.338	9.615	65.492	103.184		36.570	5.674	25.448	47.692		
ALDH1A2 + DCN	Unfavorable expression (H + H)	28.049	5.359	17.545	38.552		22.540	3.677	15.333	29.747		
	Favorable expression (L + L)	107.222	14.931	77.957	136.486	79.173	46.520	14.035	19.011	74.029	23.980	0.001
	All	89.471	12.334	65.296	113.645		33.680	6.235	21.459	45.901		
ALDH1A2 + GATA6	Unfavorable expression (H + H)	56.177	8.595	39.332	73.023		36.010	11.013	14.425	57.595		
	Favorable expression (L + L)	147.781	20.657	107.294	188.268	91.604	—	—	—	—	—	0.006
	All	107.180	13.961	79.816	134.544		54.670	11.004	33.102	76.238		
DCN + GATA6	Unfavorable expression (H + H)	33.669	5.977	21.954	45.383		27.730	1.936	23.935	31.525		
	Favorable expression (L + L)	138.157	19.002	100.913	175.400	104.488	—	—	—	—	—	0.001
	All	105.535	15.237	75.671	135.400		35.750	16.618	3.179	68.321		
GATA6 + PDGFRA	Unfavorable expression (H + H)	41.428	6.580	28.531	54.325	92.911	29.080	1.624	25.898	32.262	52.460	0.003
	Favorable expression (L + L)	134.339	21.512	92.176	176.503		81.540	—	—	—		

	All	88.740	13.317	62.639	114.841		32.490	3.629	25.376	39.604		
ALDH1A2 +	Unfavorable expression (H + H)	45.742	9.473	27.176	64.309		26.180	4.352	17.650	34.710		
PDGFRA	Favorable expression (L + L)	113.688	17.455	79.477	147.899	67.946	57.100	16.219	25.310	88.890	30.920	0.012
	All	91.677	12.710	66.765	116.589		35.750	8.355	19.374	52.126		
DCN + PDGFRA	Unfavorable expression (H + H)	33.619	5.342	23.148	44.090		27.730	3.606	20.662	34.798		
	Favorable expression (L + L)	101.756	14.105	74.111	129.401	68.137	46.520	6.052	34.657	58.383	18.790	0.001
	All	82.905	10.975	61.394	104.417		36.570	6.208	24.403	48.737		
ALDH1A2 + DCN +	Unfavorable expression (H + H + H)	30.476	6.257	18.213	42.740		25.890	2.574	20.845	30.935		
GATA6	Favorable expression (L + L + L)	153.488	21.842	110.678	196.298	123.012	—	—	—	—	—	0.001
	All	122.710	19.023	85.425	159.995		73.360	28.849	16.816	129.904		
ALDH1A2 + DCN +	Unfavorable expression (H + H + H)	28.049	5.359	17.545	38.552		22.540	3.677	15.333	29.747		
PDGFRA	Favorable expression (L + L + L)	115.898	17.873	80.867	150.930	87.850	47.800	18.911	10.734	84.866	25.260	0.001
	All	90.993	13.978	63.595	118.391		33.680	7.400	19.175	48.185		
ALDH1A2 +	Unfavorable expression (H + H + H)	46.218	11.642	23.400	69.036		27.600	1.450	24.758	30.442		
GATA6 + PDGFRA	Favorable expression (L + L + L)	146.673	24.003	99.627	193.719	100.455	—	—	—	—	—	0.005
	All	111.094	18.078	75.661	146.526		46.820	24.690	0.000	95.213		
DCN + GATA6 +	Unfavorable expression (H + H + H)	33.669	5.977	21.954	45.383		27.730	1.936	23.935	31.525		
PDGFRA	Favorable expression (L + L + L)	138.536	22.049	95.321	181.752	104.868	—	—	—	—	—	0.003
	All	98.772	16.507	66.418	131.126		33.680	4.728	24.414	42.946		
ALDH1A2 + DCN +	Unfavorable expression (H + H + H + H)	30.476	6.257	18.213	42.740		25.890	2.574	20.845	30.935		
GATA6 + PDGFRA	Favorable expression (L + L + L + L)	152.262	24.705	103.840	200.684	121.786	—	—	—	—	—	0.001
	All	116.013	20.612	75.615	156.412		35.750	28.162	0.000	90.948		

Note: The gene transcript levels were dichotomized into high and low expression with auto-selected best cutoff by the Kaplan-Meier plotter.

* Samples with high (H) expression of 1, 2, 3, and 4 genes were considered as one group, and samples with low (L) expression of 1, 2, 3, and 4 genes were considered as another group. These two groups were compared with Kaplan-Meier curves to reflect the expression of a single gene or the combined genes with prognosis. The group with long survival time was named as "favorable expression", and the group with short survival time was named as "unfavorable expression".

# Std. error, standard error; 95% CI, 95% confidence interval; Difference of estimate, mean or median estimated survival time of favorable expression – mean or median estimated survival time of unfavorable expression. Statistical tests: Kaplan-Meier survival analysis with log-rank test.
