# Supplementary figures and images for: Identification of multigene predictors of prognosis in patients with ovarian cancer
Source: iScience. 2026 Feb 6;29(3):114932. doi: 10.1016/j.isci.2026.114932 (PMC12936820; doi:10.1016/j.isci.2026.114932)

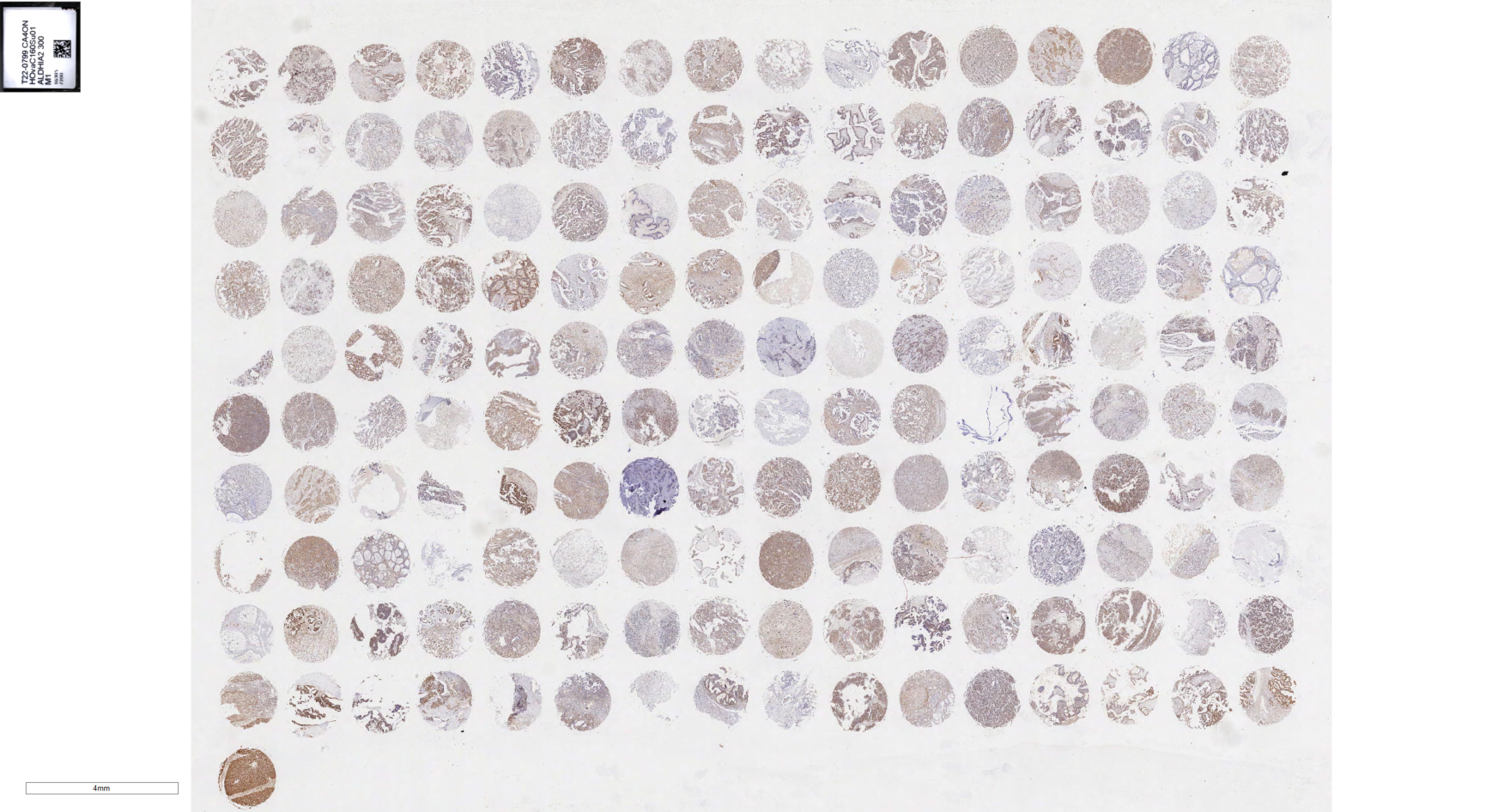

Supplement: Data S1. Information on HOvaC160Su01 — Supplemental information for HOvaC160Su01 includes an array list, an H&E staining result image, and IHC staining result images for ALDH1A2, DCN, GATA6, and PDGFRA. [file mmc3.zip › ZIP S1/HOvaC160Su01-ALDH1A2.png]

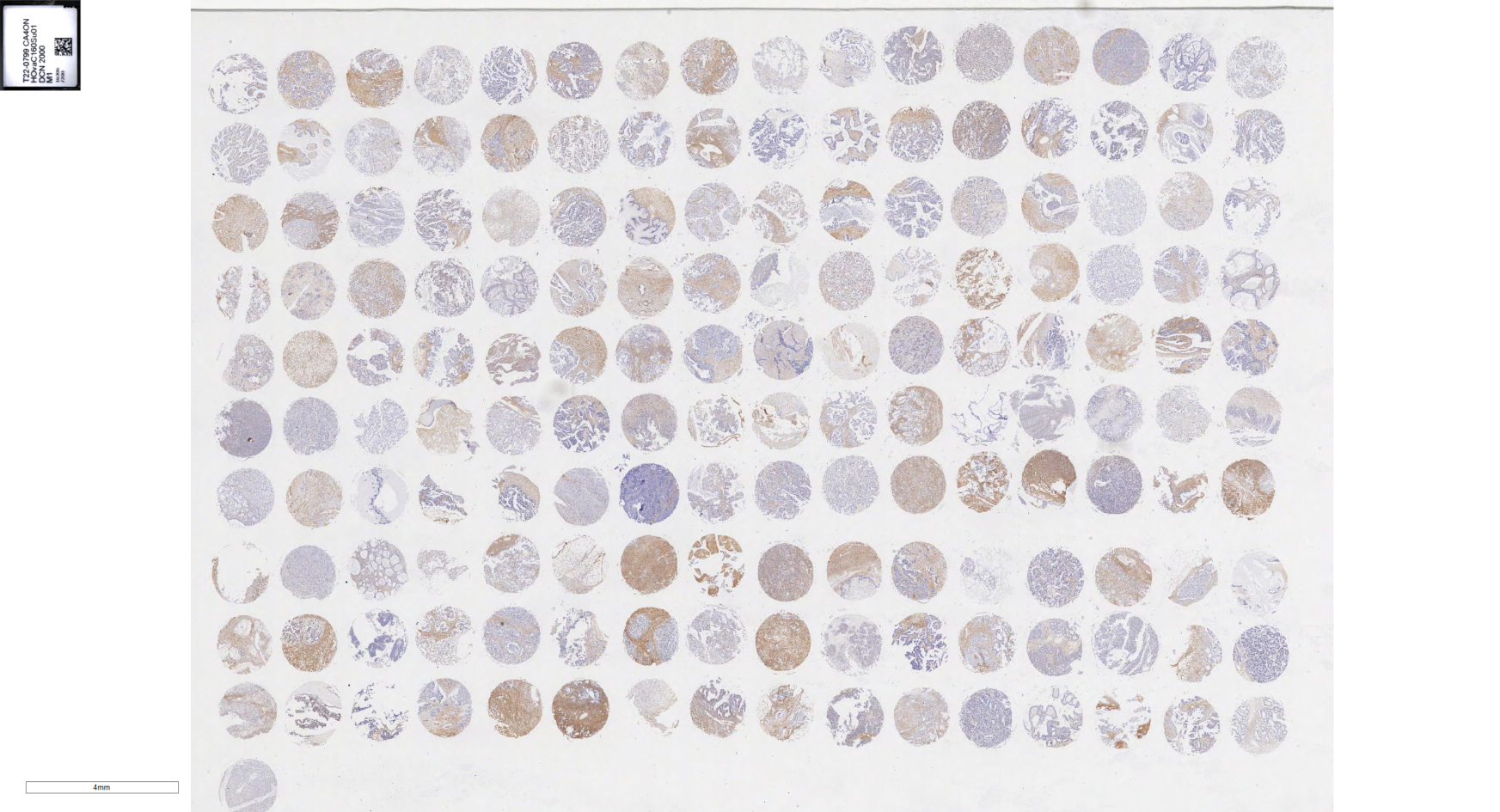

Supplement: Data S1. Information on HOvaC160Su01 — Supplemental information for HOvaC160Su01 includes an array list, an H&E staining result image, and IHC staining result images for ALDH1A2, DCN, GATA6, and PDGFRA. [file mmc3.zip › ZIP S1/HOvaC160Su01-DCN.png]

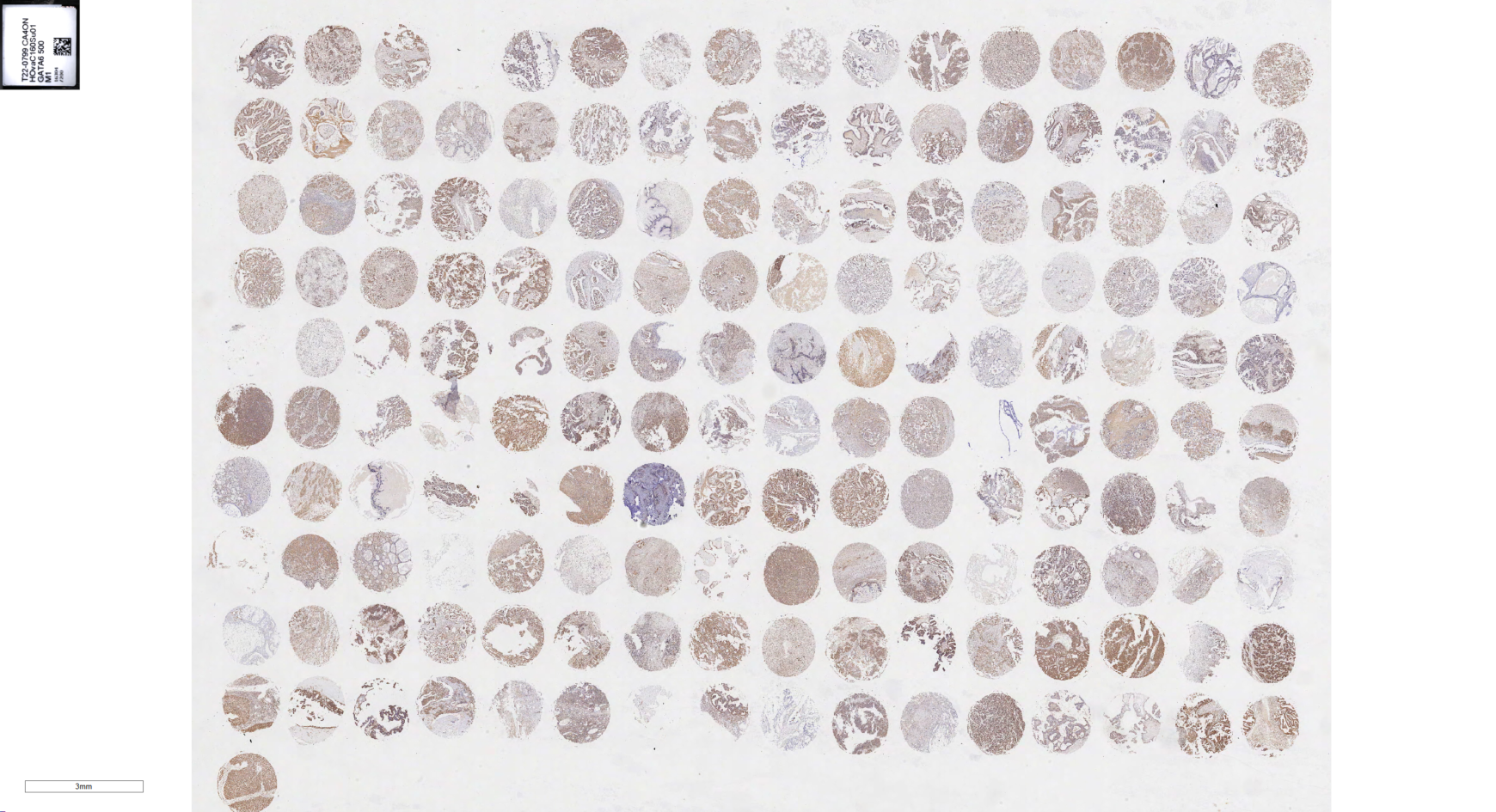

Supplement: Data S1. Information on HOvaC160Su01 — Supplemental information for HOvaC160Su01 includes an array list, an H&E staining result image, and IHC staining result images for ALDH1A2, DCN, GATA6, and PDGFRA. [file mmc3.zip › ZIP S1/HOvaC160Su01-GATA6.png]

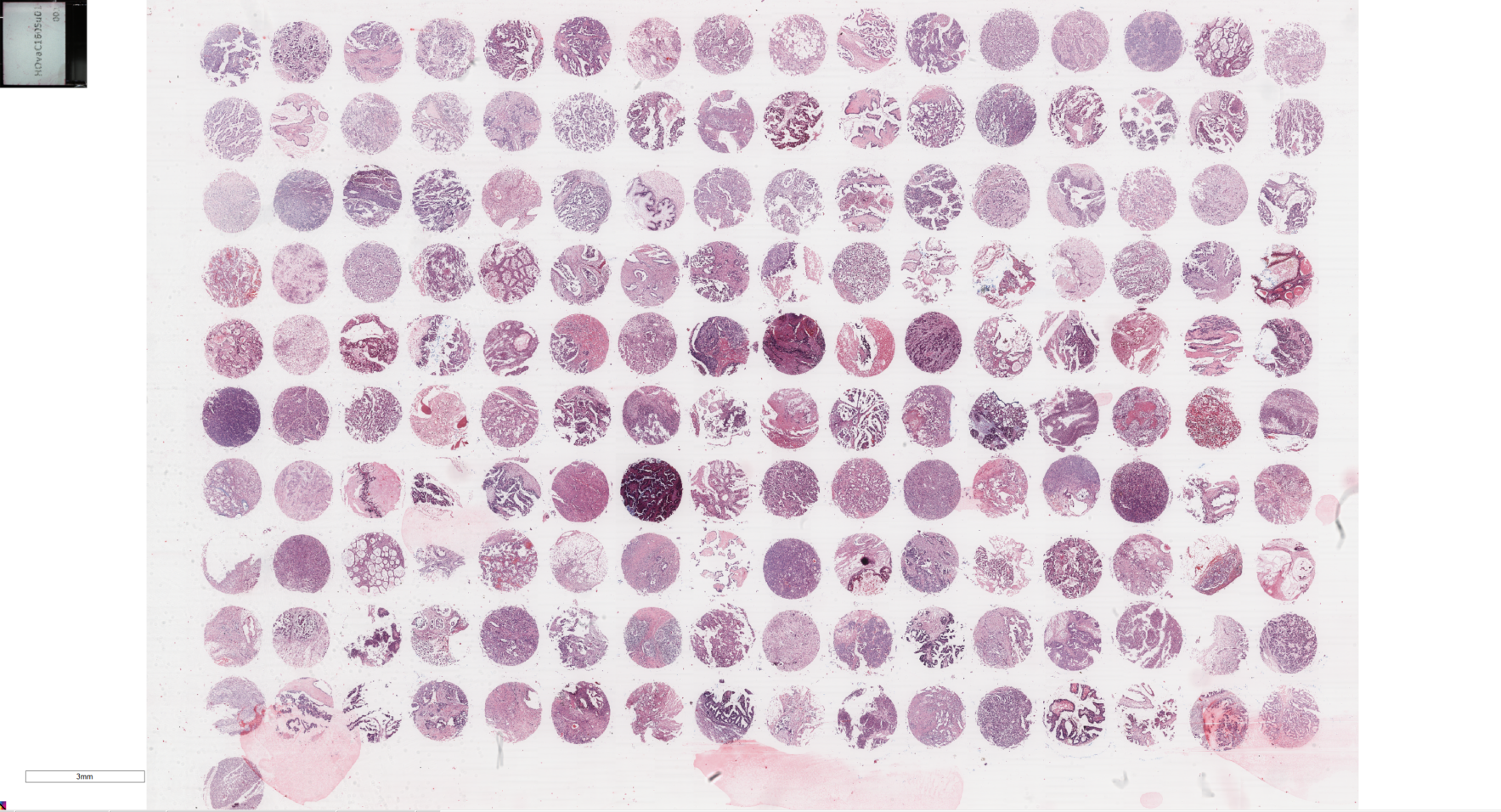

Supplement: Data S1. Information on HOvaC160Su01 — Supplemental information for HOvaC160Su01 includes an array list, an H&E staining result image, and IHC staining result images for ALDH1A2, DCN, GATA6, and PDGFRA. [file mmc3.zip › ZIP S1/HOvaC160Su01-HE staining images.png]

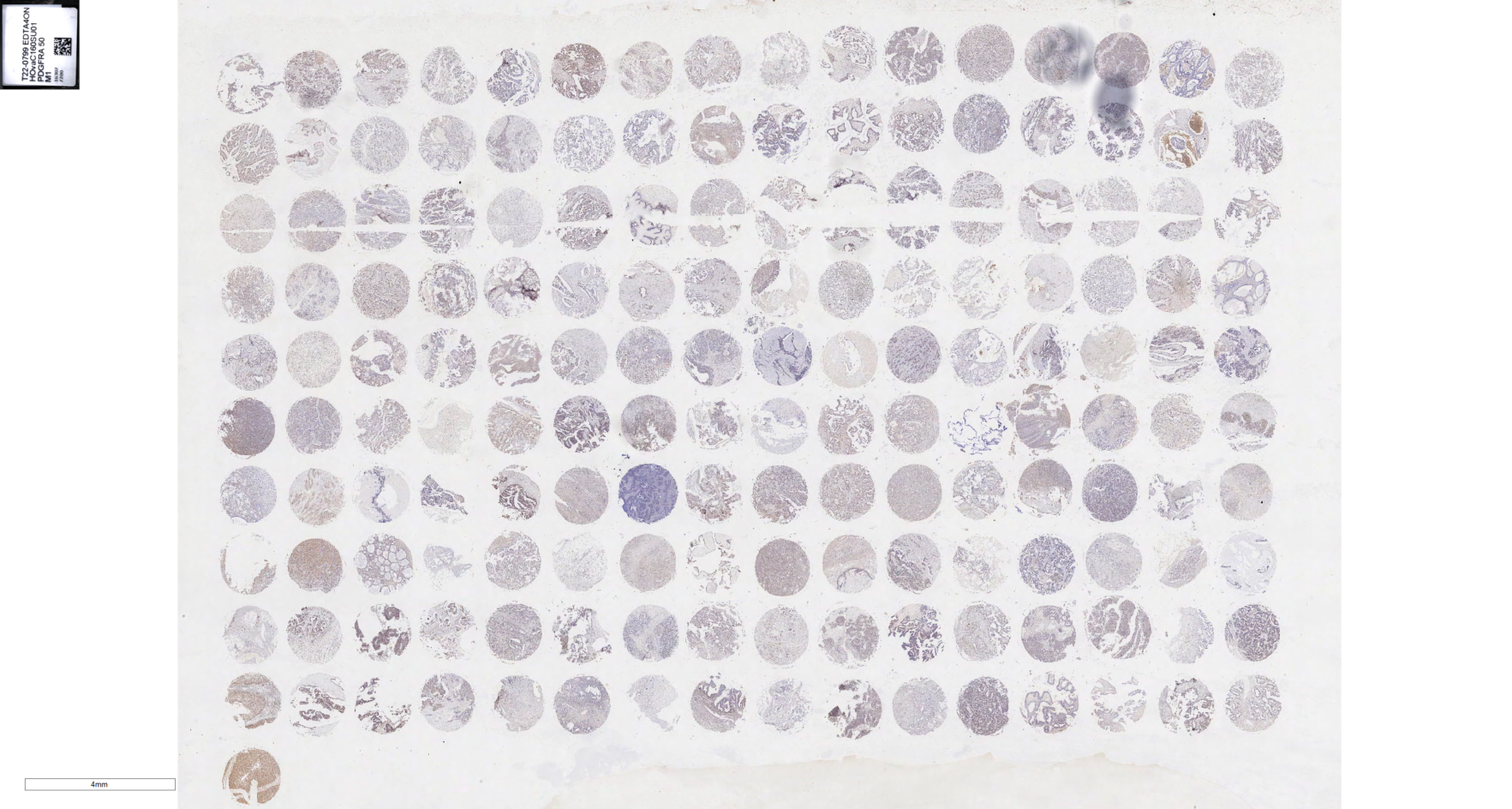

Supplement: Data S1. Information on HOvaC160Su01 — Supplemental information for HOvaC160Su01 includes an array list, an H&E staining result image, and IHC staining result images for ALDH1A2, DCN, GATA6, and PDGFRA. [file mmc3.zip › ZIP S1/HOvaC160Su01-PDGFRA.png]
